# Electrolyte- and Hydrodynamics-Controlled Potentiostatic Growth of Ag Nanodendrites on Metallic Ti for SERS Detection

**DOI:** 10.1021/acsomega.6c01538

**Published:** 2026-05-29

**Authors:** Marcos Luna-Cervantes, Erick Octavio Santos-Santiago, Diana Jiménez-Girón, José Luis Zamora-Navarro, Antonio de Jesús García-Chávez, Yuri Okolodkov, Jorge Bertín Santaella-González, Julián Hernández-Torres, Irma Yadira Izaguirre-Hernández, Pablo Thomas-Dupont, Luis Zamora-Peredo

**Affiliations:** † Centro de Investigación en Micro y Nanotecnología, Universidad Veracruzana, Av. Adolfo Ruiz Cortines 455, col. Costa Verde, Boca del Río 94294, México; ‡ Doctorado en Ciencias e Ingeniería, Universidad Autónoma de Baja California, Carretera Transpeninsular Ensenada - Tijuana 3917, col. Playitas, Ensenada 22860, México; § Instituto de Ciencias Marinas y Pesquería 27870 Universidad Veracruzana, Miguel Hidalgo 617, Río Jamapa, Boca del Río 94290, México; ∥ Facultad de Ciencias Químicas/Facultad de Ingeniería Mecánica y Ciencias Navales, Universidad Veracruzana, Av. Adolfo Ruiz Cortines 455, col. Costa Verde, Boca del Río 94294, México; ⊥ Facultad de Bioanálisis, Universidad Veracruzana, Iturbide s/n Esquina Carmen Serdán, Veracruz 91700, México; # Instituto de Investigaciones Médico Biológicas 27870 Universidad Veracruzana, Iturbide s/n entre Carmen Serdán y 20 de Noviembre, Veracruz 91700, México

## Abstract

Ag nanodendrites (AgNDs) were directly grown on nonanodized metallic Ti via potentiostatic electrodeposition (PED) to produce a simple and controllable SERS substrate. The effects of electrolyte composition and hydrodynamic conditions on dendritic growth were systematically investigated, revealing that the combined presence of a supporting electrolyte (NaNO_3_) and magnetic stirring enables sustained tip-driven growth and hierarchical branching. An optimal dendritic architecture was obtained at 2.0 V for 60 s, yielding the highest SERS enhancement. The strongest responses were found to arise from an experimentally optimal morphological regime characterized by intermediate surface coverage (∼50–65%) and well-developed secondary branching, which maximizes hotspot density while preserving interbranch gaps. Postdeposition conditioning proved critical, as mild PBS conditioning (50 mM) effectively suppressed intrinsic background signals while preserving dendritic morphology and establishing a chemically compatible interface for subsequent molecular and biomolecular interactions. The optimized AgND/Ti substrate exhibited robust analytical performance, achieving a limit of detection (*LoD*) of 3 × 10^–8^ M for Rhodamine 6G and consistent enhancement across chemically distinct dyes, including methylene blue and crystal violet. Beyond small-molecule detection, the substrate demonstrated compatibility with biomolecular SERS measurements and controlled surface functionalization. Biomolecular species produced reproducible amide-dominated spectral features, while MPA–EDC/NHS chemistry enabled stable covalent immobilization of monoclonal anti-α-fetoprotein antibodies (Ab-AFP) while maintaining a detectable and reproducible SERS response under the applied experimental conditions. Overall, these results establish clear morphology–performance and surface–functionality relationships in AgND/Ti substrates and highlight their potential as integrated platforms for molecular and biomolecular SERS-based detection.

## Graphical abstract

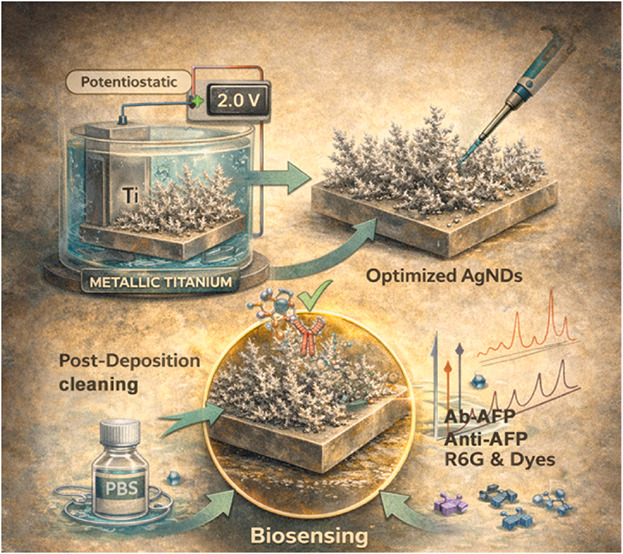

## 1 Introduction

Surface-enhanced Raman scattering (SERS) has established itself as a highly sensitive analytical technique for detecting chemical and biological analytes.^1–3,4^ Its enhancement capability arises primarily from localized surface plasmon resonance (LSPR) in metallic nanostructures, particularly within nanoscale gaps (hotspots), high-curvature regions, and fractal-like geometries, as well as from chemical enhancement mechanisms that can also occur on appropriately engineered semiconducting or nonmetallic surfaces.^5^ However, despite advances in instrumentation and data processing, the overall reliability and practical applicability of SERS remain limited by challenges in substrate fabrication.^6^ Variability in nanostructure morphology, background signals originating from surface residues, and insufficient control over growth conditions continue to hinder the development of SERS platforms suitable for demanding analytical applications.^7,8^ Moreover, in substrates that rely on hotspot-dominated amplification, such as dendritic Ag architectures,^9^ local field intensities exhibit strong spatial heterogeneity, making the evaluation of intrinsic hotspot performance highly dependent on sampling the most optically active regions.^10,11^ Beyond enhancement magnitude, the effectiveness of a SERS substrate is also strongly influenced by the interplay between nanostructure morphology and analyte size.^12^ While small molecules and molecular dyes can readily access highly confined nanoscale hotspots, larger biomolecules such as proteins and antibodies experience steric constraints that limit their access to narrow gaps and deeply confined junctions.^13,14^ As a result, SERS-active architectures that rely exclusively on highly localized hotspots may exhibit strong signals for small reporters but reduced performance for bulky analytes.^15^ Hierarchically structured and open plasmonic geometries, such as dendritic Ag architectures,^16^ offer a potential pathway to reconcile these requirements by providing multiscale hotspots with varying degrees of accessibility.^17^

Among plasmonic materials, Ag is particularly well-suited to address these morphological and accessibility constraints, as it exhibits the strongest visible-range plasmonic activity and is widely recognized for producing intense SERS enhancement.^5,18^ In particular, AgNDs have garnered considerable attention due to their ability to generate dense distributions of electromagnetic hotspots via hierarchical branching.^19,20,21^ In addition to their widespread use with molecular dyes and other small Raman reporters, dendritic Ag substrates have also been explored for biomolecular detection, including protein sensing and early immunoassay-oriented strategies.^22,23^ Their open-branched geometries and high hotspot densities facilitate analyte access to plasmonic junctions, underscoring their potential for SERS-based biosensing applications.^24^ Such structures typically emerge under nonequilibrium deposition conditions, where complex interactions between nucleation kinetics, ion transport, electric fields, and local interfacial instabilities govern growth.^25,26,27^ Precisely because of this complexity, achieving controlled dendritic morphologies remains a major challenge.

Ag nanodendrites (AgNDs) can be fabricated through a wide range of approaches, including chemical reduction, electroless growth, template-assisted synthesis, photodeposition, galvanic displacement, and electrochemical deposition.^21,28,29^ Many of these routes rely on hazardous reagents, specialized equipment, or the use of seed particles, templates, and stabilizing agents, which increase fabrication complexity and limit reproducibility.^30,31^ Electrochemical methods offer a more direct and scalable alternative;^29,32^ however, growth under constant-current conditions often leads to uncontrolled nucleation and particle agglomeration due to sustained ion depletion at the electrode interface.^33,34^ In contrast, potentiostatic electrodeposition (PED) provides a more rational strategy for dendritic growth by maintaining a fixed driving force for electron transfer, enabling finer regulation of nucleation dynamics, branching evolution, and hotspot density.^35,36^ Despite these advantages, important gaps remain in understanding how deposition parameters shape dendritic organization and SERS activity. A key limitation in current literature is the insufficient examination of how electrolyte composition influences dendritic arrangement on metallic substrates. The supporting electrolyte can modify the distribution of overpotentials, interfacial supersaturation, and overall growth stability, yet systematic comparisons remain sparse. Similarly, hydrodynamic conditions, such as agitation, are known to regulate mass transport at the electrode interface; however, their role in shaping dendritic architectures has received limited attention despite their clear relevance to diffusion-limited growth.

Additionally, background contributions from surface-bound residues remain a persistent source of spectral interference in Ag-based SERS substrates, and effective postdeposition conditioning strategies are still underexamined. Rather than requiring the complete elimination of all surface-bound species, an effective SERS substrate must ensure the suppression of spectrally active contributions under measurement conditions, while preserving interfacial compatibility with both molecular and biomolecular analytes. These challenges complicate the establishment of morphology–performance relationships, which are essential for engineering reliable SERS-active materials. Together with the sensitivity of dendritic growth to electrolyte formulation—particularly the presence or absence of a supporting electrolyte—these limitations underscore the need for deposition platforms that enable precise control over growth parameters without introducing additional complexities. However, a systematic understanding of how electrolyte composition, hydrodynamic conditions, and postdeposition surface treatment jointly govern the organization, stability, and SERS performance of AgNDs grown directly on metallic substrates under potentiostatic control remains largely unexplored.

Within this context, Ti represents an appealing base material for the direct growth of Ag nanostructures due to its chemical stability, mechanical robustness, and ability to undergo clean, well-defined electrochemical processing without the need for dielectric barrier layers, multistep oxide formation, or template-removal procedures.^37,38,39^ In contrast, oxide-based architectures, such as TiO_2_ nanotubes, typically require anodization in fluoride-containing electrolytes, prolonged processing times, multiple washing and annealing steps, and can suffer from partial delamination or structural fragility, particularly during extended electrochemical operation.^40,41,42^ Metallic Ti surfaces, by comparison, offer straightforward nucleation pathways, uniform current distribution, and direct electrical contact during PED, thereby minimizing variability associated with oxide thickness, adhesion, or defect density.^30^ These characteristics make Ag-on-Ti an experimentally accessible and controllable platform for studying morphology-dependent optical responses and for clarifying the roles of hydrodynamic conditions, electrolyte composition, and supporting-electrolyte effects.^21,43^ Notably, AgNDs and Ag nanoparticles have been predominantly grown on semiconducting substrates or on metals such as aluminum,^32,44^ copper,^45,46^ or silicon^47^ for SERS applications. In contrast, the PED of AgNDs on metallic Ti has remained largely unexplored. Taken together, these considerations highlight the need for deposition strategies that are rapid, controllable, and compatible with both molecular and biomolecular SERS detection while minimizing substrate variability and fabrication complexity.

In this work, we systematically investigate the potentiostatic growth of AgND architectures directly on metallic Ti and demonstrate how electrolyte composition, hydrodynamic conditions, and postdeposition surface treatment collectively shape dendritic organization and SERS activity. To the best of our knowledge, this study represents the first comprehensive demonstration of using nonanodized metallic Ti as a direct growth platform for AgNDs in SERS applications, enabling the decoupling of plasmonic performance from oxide-mediated or template-assisted effects. By establishing clear correlations among electrochemical growth regimes, dendritic morphology, and optical response, this work provides a reproducible and experimentally accessible framework for engineering AgND/Ti substrates. Importantly, the resulting platforms exhibit compatibility with both small-molecule Raman probes and biomolecular species, underscoring their potential relevance for future molecular and biomolecular SERS-based detection strategies.

## 2 Materials and Methods

### 2.1 Potentiostatic Electrodeposition of AgNDs

Ti foils (grade II, 5 × 10 × 0.1 mm) were ultrasonically cleaned for 10 min each in acetone (C_3_H_6_O, Karal, Mex), ethanol (C_2_H_6_O, HYCEL, Mex), and deionized water. The cleaned foils were used as cathodes in a two-electrode configuration, partially immersed to a depth of 5 mm, resulting in an effective exposed area of 5 × 5 mm per side. Two Ti foils were simultaneously employed in each deposition, with both faces exposed to the electrolyte, yielding a total effective cathodic area of 1.0 cm^2^ for current density normalization. The cathodes were positioned parallel to a platinum sheet (15 × 15 × 0.1 mm, StonyLab, USA), which served as the anode, at a fixed separation distance of 1.5 cm. Potentiostatic electrodeposition (PED) of AgNDs was carried out in two aqueous (100 mL) electrolytes: E1, containing 10 mM AgNO_3_ (Sigma-Aldrich, USA) and 100 mM NaNO_3_ (Meyer, Mexico), and E2, containing 10 mM AgNO_3_ only. Depositions were conducted at room temperature under applied potentials of 1.5, 1.75, and 2.0 V for E1, and 2.0, 3.0, 4.0, and 5.0 V for E2, using deposition times of 30, 60, 120, and 180 s with magnetic stirring at 100 rpm (PTFE-coated stir bar, 25 mm × 6 mm). A second set of experiments was performed without magnetic stirring, using applied potentials of 1.5 and 2.0 V for E1, and 3.0 and 4.0 V for E2, with the same deposition times. The current response during PED was continuously recorded in situ using a UT117C Uni-T digital multimeter.

### 2.2 Rhodamine 6G and Analyte Solutions

Rhodamine 6G (R6G, C_28_H_31_ClN_2_O_3_, Sigma-Aldrich, USA) was used as a probe molecule to assess the SERS performance of the substrates. Five aqueous solutions with concentrations ranging from 1 × 10^–3^ to 1 × 10^–7^ M were prepared using deionized water as the solvent. Following optimization, seven additional analytes were evaluated: methylene blue (MB, C_16_H_18_ClN_3_S, HYCEL, Mexico) and crystal violet (CV, C_25_H_30_ClN_3_, HYCEL, Mexico), each at 1 × 10^–5^ M; 3-mercaptopropionic acid (MPA, Sigma-Aldrich, USA) at concentrations ranging from 1 × 10^–2^ to 1 × 10^–4^ M; monoclonal anti-α-fetoprotein antibodies (Ab-AFP, MyBioSource, USA) at 5 μg/mL; α-fetoprotein antigen (AFP, MexLab, Mexico) at 50 ng/mL; bovine serum albumin (BSA, Sigma-Aldrich, USA) at 1 mg/mL; and antigliadin antibodies (Ab-Gli, Inova Diagnostics Inc., USA) at 33 U/mL. For all SERS measurements, 3 μL of each solution was deposited onto the substrates and allowed to dry under ambient conditions.

### 2.3 Surface Functionalization of AgNDs with α-Fetoprotein Antibody

The AgNDs were functionalized by depositing 10 μL of 1 × 10^–3^ M MPA onto the surface and allowing it to incubate for 1 h. The substrates were then rinsed twice with deionized water and dried at room temperature. Activation of the terminal carboxyl groups was performed by depositing 10 μL of a freshly prepared MES-buffered (pH 6.2) solution containing 10 mM 1-ethyl-3-(3-(dimethylamino)­propyl)­carbodiimide (EDC, Sigma-Aldrich, USA) and 10 mM *N*-hydroxysulfosuccinimide (NHS, Sigma-Aldrich, USA), and allowing it to react for 1 h. The substrates were then washed with 2-(N-Morpholino) ethanesulfonic acid (MES, Sigma-Aldrich, USA) buffer to remove unreacted coupling reagents. Subsequently, monoclonal anti-α-fetoprotein antibodies (Ab-AFP; MyBioSource, USA) were immobilized by depositing 10 μL of a 5 μg/mL Ab-AFP solution onto the activated surface, followed by a 30 min incubation and a final rinse with phosphate-buffered saline at 1 × 10^–2^ (PBS, Sigma-Aldrich, USA).

### 2.4 Raman Spectroscopy, Morphological, and Elemental Characterization

SERS measurements were carried out using a confocal Raman microscope (Thermo Scientific DXR) equipped with a 780 nm excitation laser. Unless otherwise stated, spectra were acquired using a 10× objective at a laser power of 24 mW, yielding an approximate spot size of ∼3 μm. The 780 nm excitation wavelength was selected to minimize fluorescence background and prevent photoinduced reactions or degradation of the analytes, which are more likely to occur at shorter wavelengths.^48^

For the limit of detection (*LoD*) and small-molecule experiments, a 50× objective was employed, resulting in a reduced spot size of approximately ∼1 μm to enhance spatial confinement and local hotspot probing.^49,50^ Each spectrum corresponds to the average of five accumulations with an integration time of 5 s per scan. For each deposition condition, Raman spectra were collected from at least ten distinct locations on the substrate to account for spatial heterogeneity.

The morphology of the AgNDs was examined using a benchtop scanning electron microscope (JCM-6000, JEOL, USA) operated at 15 kV in secondary-electron mode with an 11 mm working distance. Elemental composition was examined by field-emission scanning electron microscopy (FESEM-EDS, JEOL JSM-7600F). For statistical analysis, morphological parameters were quantified from SEM images by performing at least 30 independent measurements per sample using ImageJ (v1.53k). The analyzed features included the primary trunk length, secondary branch length, and surface coverage.

### 2.5 Quantitative SERS Analysis and Enhancement Factor Calculation

Quantitative SERS analysis was performed using the Raman band at 1507 cm^–1^ of R6G as the analytical signal, selected for its high intensity and spectral isolation on Ag-based SERS substrates.^51^ Calibration measurements were conducted on the optimized AgND/Ti substrates (2.0 V, 60 s, magnetic stirring, followed by PBS conditioning) using R6G concentrations ranging from 1 × 10^–3^ to 1 × 10^–7^ M.

For each concentration, Raman spectra were acquired from at least ten different locations under identical instrumental conditions. The mean peak intensity was used to construct the calibration curve as a function of log10­(*C*), where *C* is the analyte concentration. This approach accounts for the intrinsic spatial variability of hotspot-dominated SERS substrates while enabling statistically meaningful quantitative analysis.^52,53^

The NO-SERS reference was obtained by depositing the same R6G solution onto the Ti substrate in the absence of AgNDs and measuring it under identical acquisition conditions. In this work, this condition is defined as *I*_*ref*_ and is used as the reference signal for both limit-of-detection (*LoD*) estimation and analytical enhancement factor calculations (*AEF*).

The *LoD* was determined using the criterion *I*_*LOD*_ = *I*_*ref*_ + 3*SD*_*ref*_, where correspond to the mean intensity and standard deviation of the NO-SERS reference signal, respectively.^54^ This definition ensures that the estimated *LoD* reflects the true plasmonic contribution of the AgND substrate relative to the nonenhancing background.

The *AEF* was calculated according to eq 1,^55^ where *I*_*SERS*_ corresponds to the Raman intensities measured on the AgND/Ti substrate. *C*_*SERS*_ and *C*_*ref*_ represent the corresponding analyte concentrations. For AEF calculations, *C*_*ref*_ was fixed at 1 × 10^–3^ M, and all measurements were performed under identical instrumental conditions to ensure consistency and comparability.

1 $$AEF=\left(\frac{C_{ref}}{C_{SERS}}\right)\left(\frac{I_{SERS}}{I_{ref}}\right)$$

## 3 Results and Discussion

### 3.1 Potentiostatic Growth Regimes and Current–Time Behavior

The potentiostatic growth of AgNDs on metallic Ti was first analyzed through the evolution of current density as a function of deposition time under different electrolyte compositions, applied potentials, and hydrodynamic conditions (Figure 1). Figure[Fig fig1] [Fig fig1]a and 1b shows the current–time profiles obtained under magnetic stirring for E1 and E2, respectively, while Figure 1c and 1d corresponds to the same electrolytes without magnetic stirring.

**Figure 1:**
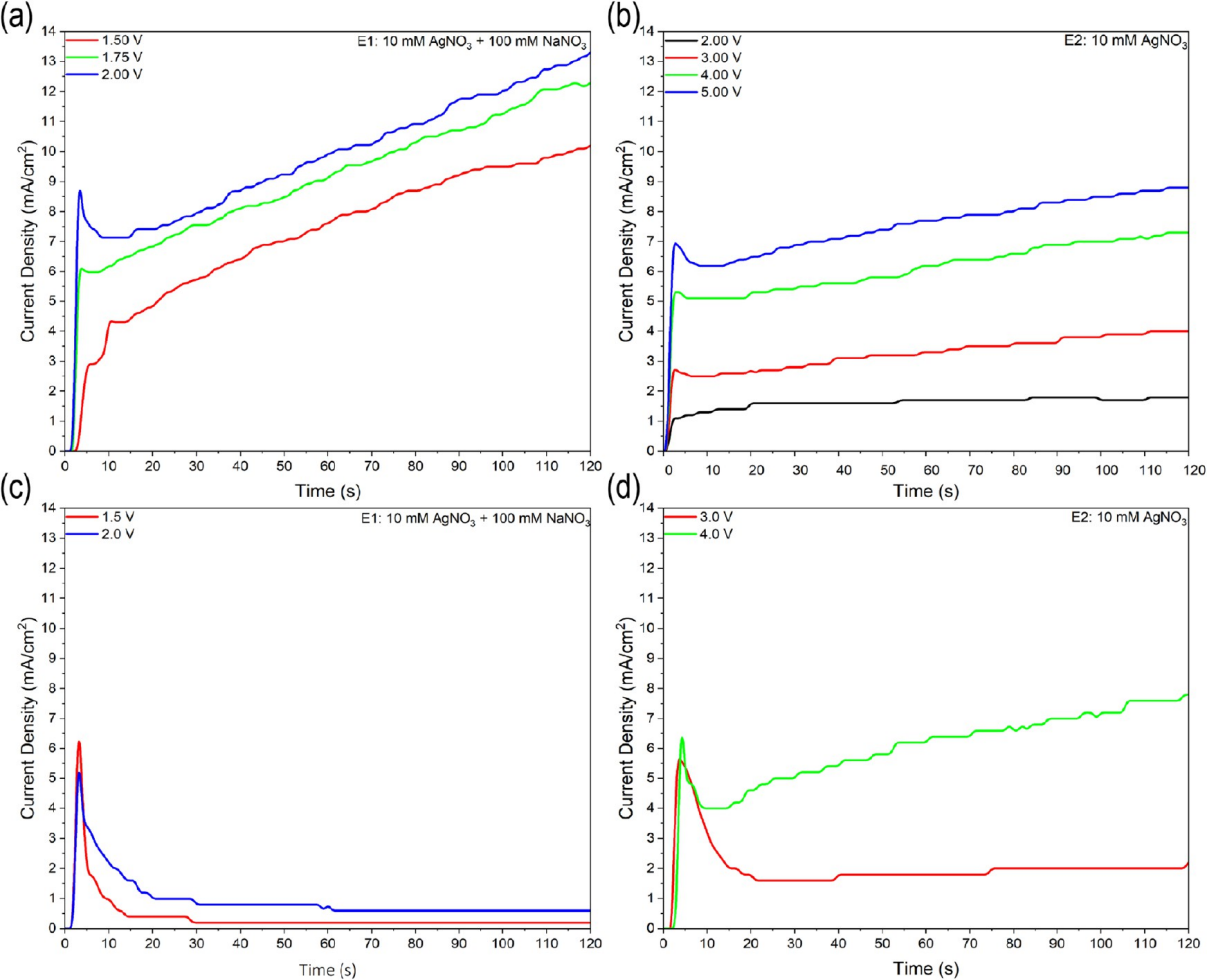
Current density–time profiles during PED of AgNDs on metallic Ti under different electrolyte compositions and hydrodynamic conditions: (a) E1 (10 mM AgNO_3_ + 100 mM NaNO_3_) with magnetic stirring, (b) E2 (10 mM AgNO_3_) with magnetic stirring, (c) E1 without stirring, and (d) E2 without stirring.

Under magnetic stirring, E1 exhibits a well-defined and stable current–time response across all investigated potentials (1.5–2.0 V), as shown in Figure 1a. After a short initial transient associated with double-layer charging and rapid nucleation,^56^ the current density evolves into a quasi-monotonic increase with time. This gradual rise in current density is consistent with a progressive increase in electrochemically active surface area, indicative of sustained dendritic growth rather than planar deposition.^57^

From an electrochemical perspective, the observed current–time behavior can be interpreted within the framework of potentiostatic metal electrodeposition. The initial current transient is associated with double-layer charging and early-stage nucleation processes, followed by a growth regime likely governed by ion transport toward the electrode surface. Under conditions where mass transport is sustained (i.e., in the presence of both supporting electrolyte and convective flow), the progressive increase in current density is consistent with continuous surface area amplification and tip-enhanced growth. In contrast, the rapid current decay observed under static conditions is consistent with diffusion-limited regimes, where local Ag^+^ depletion suppresses sustained deposition. These interpretations are well-supported by established electrochemical models and recent studies on metal electrodeposition and dendritic growth under potentiostatic conditions.^36,58–60,61^

The separation between the current–time curves is preserved throughout the deposition window, following the order 2.0 V > 1.75 V > 1.5 V, indicating effective potentiostatic control and homogeneous ion transport promoted by the presence of NaNO_3_ as the supporting electrolyte. In contrast, deposition in E2 under magnetic stirring (Figure 1b) displays a markedly different behavior. Although higher applied potentials (2.0–5.0 V) lead to increased current densities, the corresponding *j*–*t* curves exhibit more pronounced transients and step-like features. The absence of a supporting electrolyte results in enhanced sensitivity to local ion depletion and electric-field gradients, leading to less smooth current evolution despite continuous agitation.^62^ These features indicate that, while convective transport partially mitigates mass-transfer limitations, E2 growth remains more strongly influenced by localized concentration gradients, particularly at higher applied potentials.

The role of hydrodynamics becomes more evident in the absence of magnetic stirring. For E1 without magnetic stirring (Figure 1c), the current density exhibits a sharp initial peak followed by a rapid decay toward low, quasi-steady values, regardless of the applied potential (1.5–2.0 V). This behavior is characteristic of diffusion-limited deposition, where rapid Ag^+^ consumption near the electrode surface leads to local depletion and suppresses sustained growth.^58^ In this regime, the influence of the applied potential is significantly reduced, indicating that mass transport, rather than the electrochemical driving force, dominates the deposition process.

A similar but more accentuated trend is observed for E2 without magnetic stirring (Figure 1d). At 3.0 V, the current rapidly decays after the initial transient and stabilizes at low values, reflecting severe transport limitations in the absence of both convective flow and supporting electrolyte. At 4.0 V, a partial recovery and gradual increase in current density are observed over time; however, the overall response remains less stable and more irregular than under stirred conditions. This behavior highlights the compounded effect of limited ion mobility and the absence of supporting electrolyte in destabilizing the potentiostatic growth regime. This observation is supported by the current–time behavior, which shows diffusion-limited decay and diminished voltage sensitivity in E1 without stirring (Figure 1(c)), and two clearly distinct regimes in E2 at 3.0 and 4.0 V (Figure 1d).

Overall, the current–time analysis presented in Figure 1 suggests that sustained dendritic growth on metallic Ti requires the combined presence of convective mass transport and a supporting electrolyte.^63^ Magnetic stirring promotes continuous Ag^+^ replenishment at the electrode interface,^64^ while NaNO_3_ stabilizes the electric field and suppresses abrupt concentration gradients.^65,66,67^ Together, these factors enable controlled potentiostatic growth behavior, characterized by progressive increases in current density, indicative of dense, hierarchically branched AgND architectures.

Although techniques such as cyclic voltammetry and chronoamperometry can provide complementary insights into redox kinetics, the present analysis focuses on current–time responses acquired under the actual PED conditions used for substrate fabrication, as these directly capture the operative growth regimes under practical deposition conditions. These electrochemical growth regimes provide the basis for the morphological and SERS performance analyses discussed in the following sections.

### 3.2 Effect of Electrolyte Composition on Dendritic Morphology

Figure 2 shows the pronounced influence of electrolyte composition on the morphology and temporal evolution of AgNDs grown potentiostatically on metallic Ti under magnetic stirring at a fixed applied potential of 2.00 V. Figure 2a–c corresponds to deposits obtained from E1, while Figure 2d–f shows the corresponding structures formed in E2 at deposition times of 30, 60, and 180 s. As established by the current–time analysis in Section 3.1, the presence of a supporting electrolyte stabilizes the electric field and mitigates concentration gradients, enabling sustained tip-driven growth. This electrochemical regime is consistent with the dendritic morphologies observed in Figure 2.

**Figure 2:**
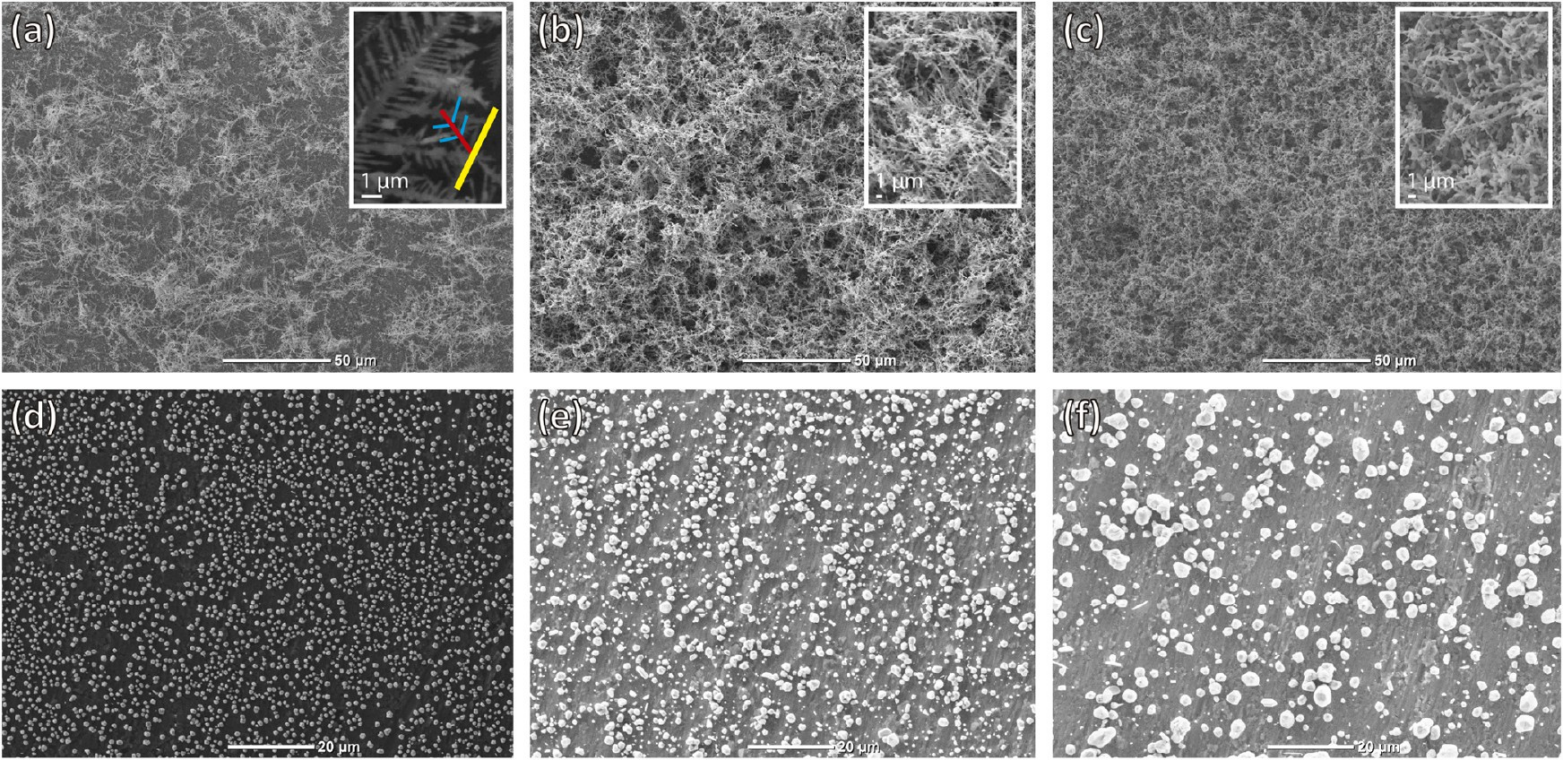
SEM images of Ag deposits grown potentiostatically at 2.0 V under magnetic stirring from aqueous electrolytes: (a–c) E1 (10 mM AgNO_3_ + 100 mM NaNO_3_) after 30, 60, and 180 s, showing the progressive development of interconnected dendritic architectures; (d–f) E2 (10 mM AgNO_3_) after 30, 60, and 180 s, exhibiting particle-dominated growth with increasing aggregate size over time. (a) Inset shows a schematic representation of branched structures composed of primary trunks (yellow line) decorated with secondary (red line) and tertiary (blue line) branches.

In the presence of the supporting electrolyte (E1), Ag growth proceeds through the rapid formation of interconnected AgND architectures, already evident at short deposition times (Figure 2a). At 30 s, a sparse but continuous network of branched AgND structures covers the Ti surface, composed of primary trunks decorated with secondary and tertiary branches (Figure 2a, inset). The characteristic branch length at this stage is on the order of 11.3 ± 1.9 μm, with lateral extension lengths of approximately 1.9 ± 0.8 μm. As the deposition time increases to 60 s (Figure 2b), the AgND network becomes denser and more uniformly distributed, with a clear increase in branch multiplicity and junction density, reflecting sustained tip growth and repeated branching rather than simple thickening. After 180 s (Figure 2c), the Ti surface is covered by a highly interconnected AgND framework; however, higher-magnification inspection reveals a gradual loss of fine branch definition, accompanied by lateral coalescence and partial fusion of neighboring branches. This evolution indicates a transition from tip-dominated growth toward a coarsening regime, in which continued Ag deposition promotes branch thickening rather than the formation of new high-order branches.^68,69^ Despite this local aggregation, the AgND architecture remains continuous and highly interconnected, preserving a multiscale porous network across the Ti surface.^16,70^ At this stage, the high dendrite density and structural overlap hinder the clear distinction between primary trunks and secondary branches, preventing reliable quantitative measurement of trunk length while still indicating increased surface coverage and structural coarsening.

In contrast, Ag deposition from E2, which lacks a supporting electrolyte, results in a fundamentally different growth mode despite identical hydrodynamic conditions. At 30 s (Figure 2d), the surface is populated by discrete Ag nanoparticles distributed across the Ti substrate, with an average particle diameter of 1.2 ± 0.6 μm and no evidence of interconnection or directional branching. With increasing deposition time to 60 s (Figure 2e), particle density remains high, while individual nanoparticles grow in size, reaching average diameters of 3.2 ± 1.3 μm. Prolonged deposition to 180 s (Figure 2f) leads primarily to further particle coarsening, with average diameters increasing to 4.2 ± 1.1 μm, accompanied by partial aggregation but without the emergence of dendritic or fractal connectivity.^71^

These distinct morphologies are consistent with the electrochemical growth regimes inferred from the current–time analysis (Section 3.1), where differences in ion transport and electric field distribution are likely to govern the transition between dendritic and particle-dominated growth. Upon application of the cathodic bias, Ag deposition proceeds via the reduction of Ag^+^ ions^27^ at the Ti surface (eq 2):

2 $${\mathrm{Ag}}^{+}+e^{-}\to {\mathrm{Ag}}^{0}$$

with charge balance maintained by anodic oxygen evolution at the Pt counter electrode (eq 3):

3 $$2H_{2}O\to O_{2}+4H^{+}+4e^{-}$$

While these electrochemical reactions are identical for both electrolytes, the presence or absence of a supporting electrolyte plays a key role in governing the effective growth regime by modulating the spatial distribution of the electric field and the local availability of Ag^+^ at the electrode interface. In E1, enhanced ionic conductivity and reduced Ohmic drop enable sustained Ag^+^ flux toward protruding features, promoting diffusion-limited instabilities that drive repeated branching and the formation of hierarchically organized AgND architectures. In contrast, the lower solution conductivity in E2 intensifies transport limitations and electric field localization, favoring localized nucleation and isotropic particle growth over sustained directional branching. Consequently, the temporal evolution in E2 is dominated by nanoparticle enlargement and coarsening rather than by the development of interconnected AgND networks, even under convective conditions. Overall, Figure 2 demonstrates that the electrolyte composition alone can drive a transition between dendritic and particle-dominated morphologies under otherwise identical potentiostatic and hydrodynamic conditions. These distinct growth modes establish markedly different surface textures and characteristic length scales, which are expected to play a decisive role in the subsequent SERS performance of AgND/Ti substrates.

### 3.3 Role of Hydrodynamic Conditions under Diffusion-Limited Dendritic Growth

The influence of hydrodynamic conditions on Ag growth was further examined by suppressing magnetic stirring and analyzing the resulting morphologies obtained from E2 under potentiostatic control (Figure 3). Consistent with the electrochemical behavior discussed in Section 3.1, suppression of convective mass transport likely limits Ag^+^ replenishment at the electrode interface, leading to diffusion-dominated growth modes and allowing a qualitative assessment of the electric field strength required to sustain dendritic growth.

**Figure 3:**
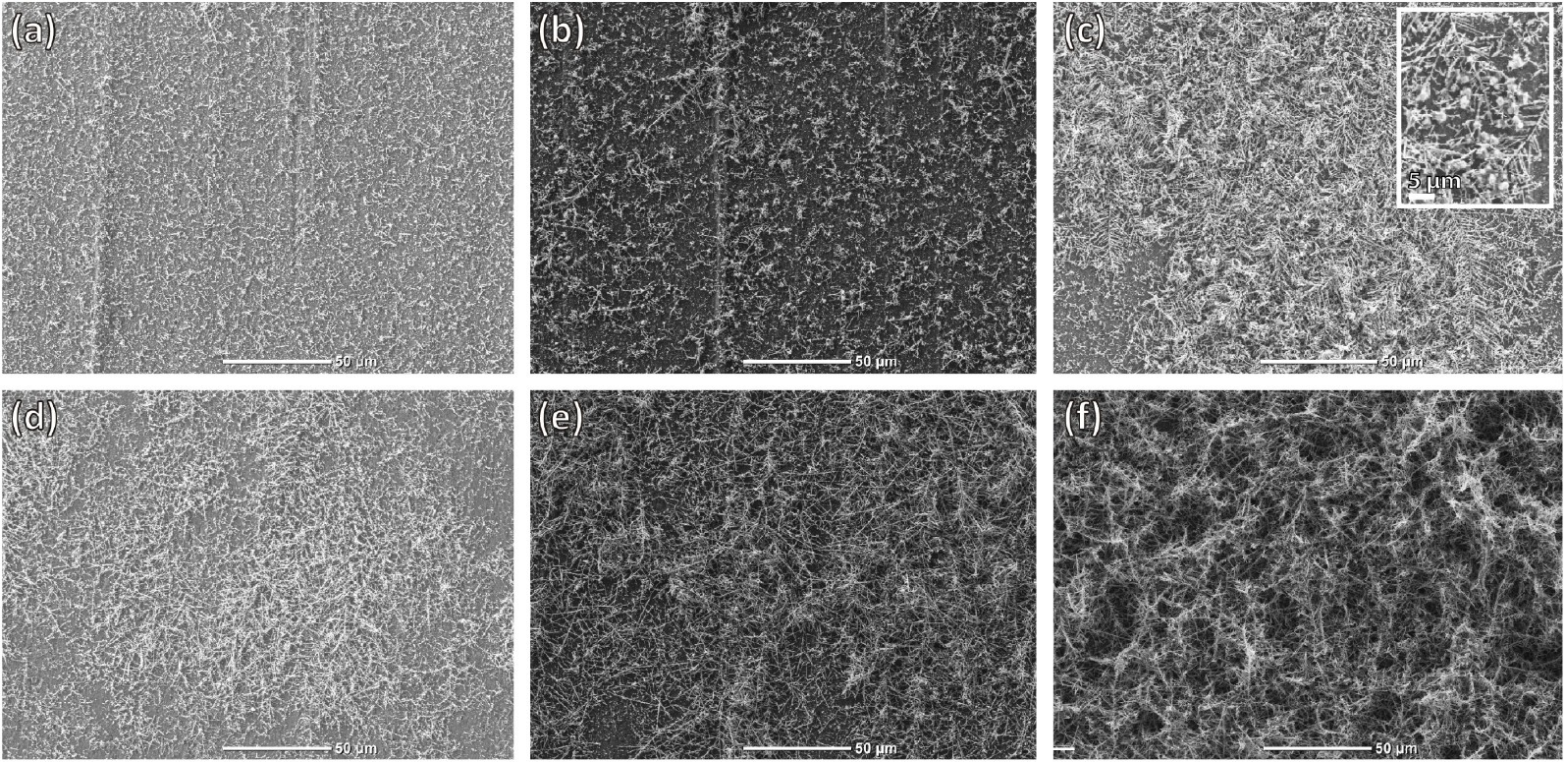
SEM images of AgNDs deposited from E2 (10 mM AgNO_3_) without magnetic stirring at (a–c) 3.0 V and (d–f) 4.0 V for deposition times of 30, 60, and 180 s, respectively. The inset in (c) highlights the presence of nanoparticle agglomerates and spike-like features formed under diffusion-limited conditions.

At an applied potential of 3.0 V without magnetic stirring (Figure 3a–c), Ag deposition produces heterogeneous surface textures characterized by the coexistence of isolated nanoparticles, small agglomerates, and only sporadic elongated features. At short deposition times (30 s), the surface is dominated by discrete particles and compact clusters, with minimal evidence of branching. With deposition times of 60 and 180 s, particle coarsening becomes more pronounced, yielding irregular aggregates, while extended dendritic connectivity remains suppressed. These observations suggest that, under diffusion-limited conditions, an applied potential of 3.0 V is insufficient to overcome Ag^+^ depletion at the electrode interface, establishing a particle-dominated growth regime that defines the lower electric-field threshold for dendritic propagation in the absence of convective transport.^33,72,73^

In contrast, increasing the applied potential to 4.0 V without magnetic stirring results in a distinct growth regime (Figure 3d–f). Even at short deposition times, the surface becomes populated by elongated, needle-like Ag structures extending radially from nucleation sites. With prolonged deposition, these features evolve into continuous AgND networks composed of markedly thinner branches than those formed under convective conditions (Figure 4). The absence of discrete nanoparticle agglomerates at 4.0 V indicates that the enhanced electric-field driving force partially compensates for the lack of convective Ag^+^ replenishment by promoting localized ion migration toward protruding features and field-enhanced tip growth under diffusion-limited conditions.^33,63^ ,^74^ It is important to emphasize that the emergence of ultrathin dendritic features at elevated potentials under static conditions does not contradict the critical role of hydrodynamic control. Rather, high applied potentials partially compensate for mass-transport limitations by amplifying local electric-field gradients and Ag reduction rates at protruding sites. However, such voltage-driven dendritic growth remains less stable and reproducible than that achieved under convective conditions, which consistently sustain robust tip-dominated growth across extended time scales.

**Figure 4:**
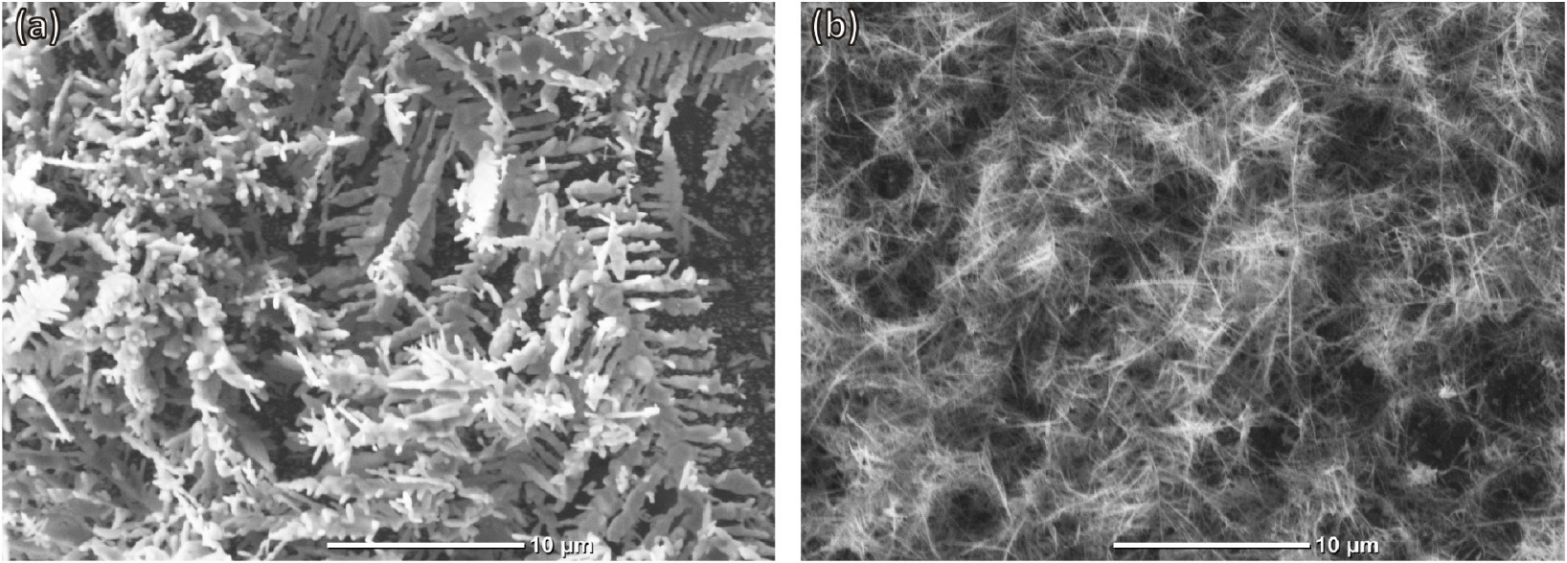
SEM images of AgNDs electrodeposited from E2 (10 mM AgNO_3_) at 4.0 V for 180 s under (a) magnetic stirring and (b) static conditions. Magnetic agitation promotes the formation of well-developed, interconnected dendritic architectures, whereas deposition without stirring yields thinner, spike-like features with reduced branching.

The impact of hydrodynamic conditions on dendritic morphology is further illustrated by the direct comparison shown in Figure 4, which examines AgNDs grown from E2 at 4.0 V for 180 s under magnetic stirring and without magnetic stirring. Under magnetic stirring (Figure 4a), growth proceeds through the development of well-defined AgND architectures composed of elongated primary trunks and abundant secondary branching, forming an open and highly interconnected network. In contrast, deposition without magnetic stirring (Figure 4b) yields thinner, needle-like features with reduced lateral branching and a more heterogeneous spatial distribution. This pronounced morphological divergence, obtained under identical electrochemical driving force and deposition time, indicates that convective mass transport plays a key role in sustaining reproducible, hierarchically branched AgND architectures. Without magnetic stirring, Ag^+^ depletion near the electrode interface limits lateral growth and branching, favoring sparse, spike-like structures rather than fully developed AgND networks. These observations are fully consistent with the current–time behavior discussed in Section 3.1 and underscore the need for controlled hydrodynamic conditions to achieve reliable potentiostatic growth of AgNDs.

The suppressive effect of diffusion-limited conditions on dendritic growth is further evidenced by the morphologies obtained from E1 without magnetic stirring (Figure 5). At all investigated deposition times, the Ti surface is predominantly populated by discrete Ag nanoparticles and irregular agglomerates, with only sporadic formation of elongated features. At 30 and 60 s [Figures 5a and 5b], deposition is dominated by homogeneous nucleation followed by particle growth, yielding dense distributions of nanoscale and submicrometer aggregates without extended connectivity. After prolonged deposition (180 s), the surface remains largely particle-dominated (Figure 5c), as highlighted by the inset, where Ag deposits appear primarily as coalesced nanoparticles and compact clusters. Only a limited number of spike-like elongated features are observed, exhibiting minimal secondary branching and no evidence of hierarchical dendritic organization. These observations demonstrate that, even in the presence of a supporting electrolyte, the absence of convective transport suppresses the emergence of interconnected AgND architectures, highlighting the important role of hydrodynamic control in enabling sustained dendritic growth under potentiostatic conditions.

**Figure 5:**
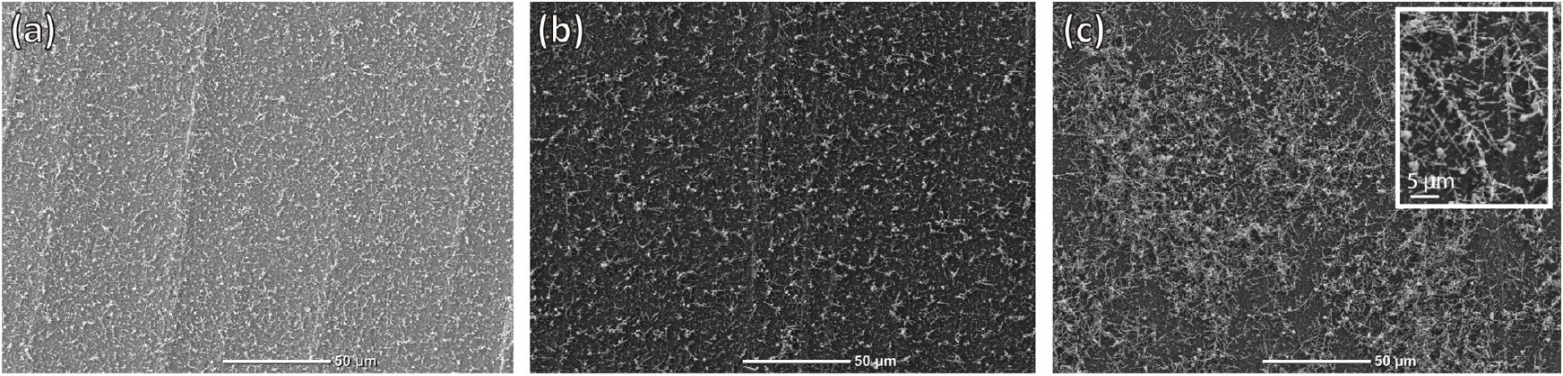
SEM images of Ag deposits formed from E1 (10 mM AgNO_3_ + 100 mM NaNO_3_) without magnetic stirring at 2.0 V for deposition times of (a) 30 s, (b) 60 s, and (c) 180 s. The inset in (c) shows the dominance of nanoparticle agglomerates and the absence of well-defined dendritic branching at longer deposition times.

### 3.4 Influence of Postdeposition Surface Conditioning

SERS measurements are highly sensitive not only to the target analyte but also to any residual species present on the plasmonic surface. In practice, insufficient postsynthesis conditioning can lead to misleading spectral interpretations, particularly when probing molecules containing common functional groups such as C=C (∼1605 cm^–1^), N–H (∼1390–1405 cm^–1^), C–N (∼1130 cm^–1^), C–H (∼1040 cm^–1^), C–O (∼810 cm^–1^), or NH_2_ (∼750 cm^–1^). These vibrational modes are frequently observed in molecular dyes^55^ (e.g., methylene blue) as well as in biomolecules such as proteins and antibodies.^75^ Consequently, residual background signals arising from the SERS substrate itself may be misassigned to the analyte, leading to false-positive detection. Figure S1 (Supporting Information) shows the Raman spectrum acquired from AgNDs immediately after electrodeposition, without any postdeposition surface conditioning. A rich set of intense bands is observed across the full spectral range, evidencing a strong intrinsic background signal. These features are consistent with surface-bound nitrate/nitrite species and carbonaceous residues remaining from the aqueous electrodeposition process. During electrodeposition, these species are readily incorporated or adsorbed onto the Ag surface through electrostatic interactions and partial chemisorption, particularly at low-coordination sites such as dendrite tips and junctions, where local electric fields and surface charge density are highest. Similar background contributions have been previously reported for Ag nanostructures;^8,49,76^ however, systematic efforts to suppress this signal have remained limited.

To address this issue, a series of postdeposition conditioning protocols were systematically evaluated. Rinsing with deionized water, widely reported as a postsynthesis/postdeposition step for Ag nanostructures, often implemented in repeated cycles,^77–80^ was first examined. Additional treatments, including exposure to a carbonated solution^77^ and polarity inversion,^81^ were explored as alternative strategies to disrupt weakly bound ionic residues and adsorbed surface species generated during synthesis. Finally, conditioning with phosphate-buffered saline (PBS) was investigated based on prior reports,^82^ which demonstrated the chemical stability of silver nanoislands in various buffer environments, including PBS. This suggests that PBS can serve as an effective conditioning medium capable of suppressing surface-related spectral contributions while preserving the structural integrity of the metallic nanostructure and maintaining compatibility with biomolecular systems.

Figure 6a compares the Raman spectra obtained after applying these different conditioning strategies. Although all treatments modify the spectral profile to some extent, none of the approaches based on deionized water (red line), carbonated solution (blue line), or polarity inversion (orange line) fully eliminate the background signal (black line). Persistent bands remain throughout the spectrum, indicating that weakly bound surface contaminants are strongly retained by the highly active AgND surface. In some cases, such as polarity inversion, additional spectral features and intensity amplification are observed, suggesting that electrochemical perturbation may redistribute or even enhance certain surface-bound species rather than remove them. Similarly, carbonated solutions partially attenuate selected bands but introduce broad features associated with carbonate-related species, resulting in a complex and nonflat baseline. In contrast, a markedly different behavior is observed when PBS is used as the postdeposition conditioning medium (magenta line). The PBS-conditioned surface exhibits a nearly featureless baseline across the entire Raman window, with the complete suppression of bands previously associated with nitrate, nitrite, and carbon-related residues. This result demonstrates that PBS effectively suppresses surface-related spectral contributions associated with residual species, yielding a spectrally clean plasmonic substrate without introducing additional Raman-active contaminants. In addition to spectral cleanliness, the effectiveness of postdeposition conditioning must be evaluated in terms of its compatibility with downstream biomolecular applications. In practical SERS-based biosensing, the substrate surface must not only be free of interfering background signals but also provide a chemically suitable interface for biomolecular adsorption and functionalization. In this regard, PBS serves a dual role: it suppresses surface-related spectral contributions while simultaneously establishing an environment inherently compatible with antibody–antigen systems. The chemical stability of silver nanostructures in PBS has been previously reported, further supporting its use as a conditioning medium that preserves structural integrity while enabling biofunctionalization.

**Figure 6:**
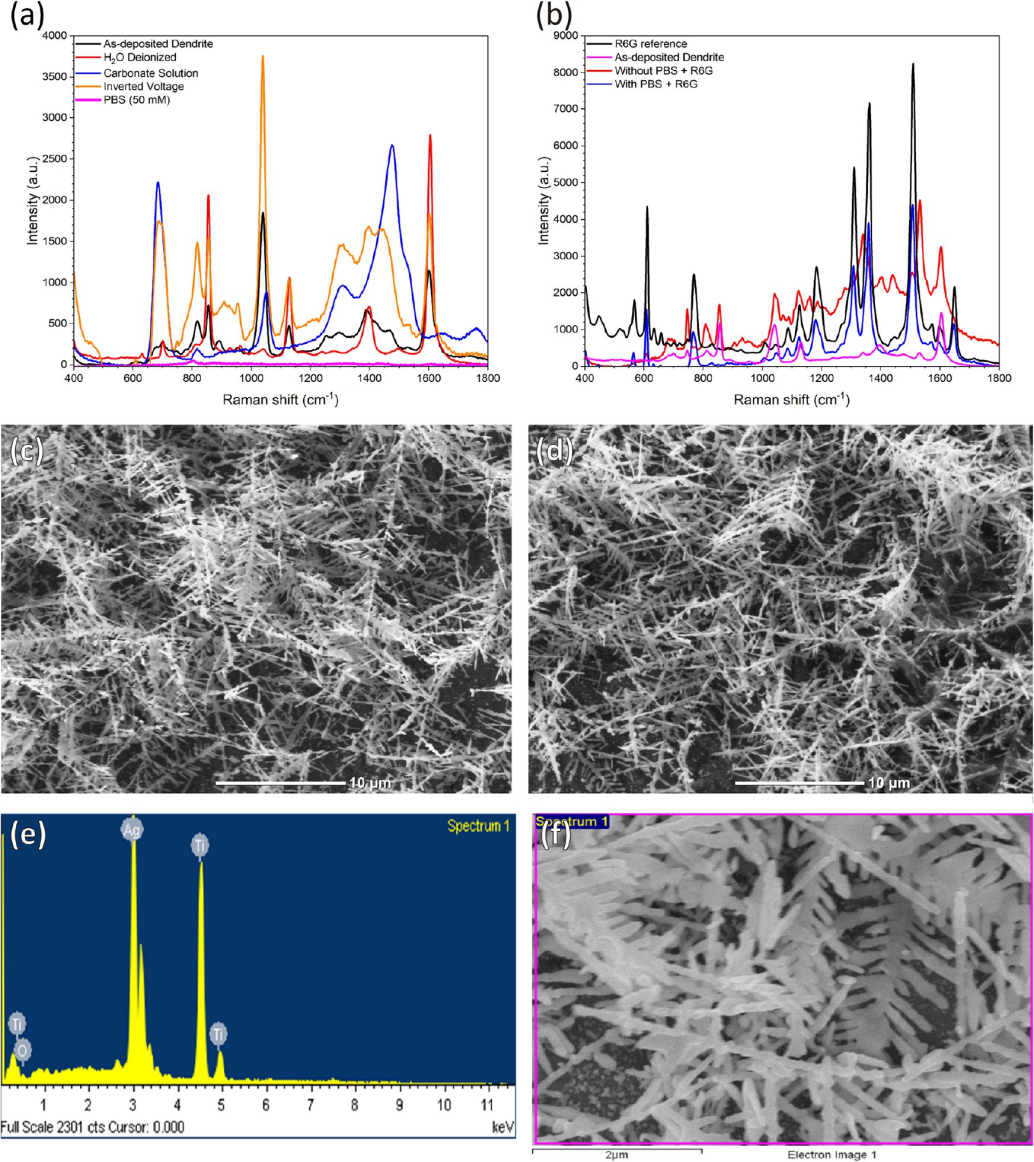
(a) Comparison of Raman background signals obtained after different postdeposition conditioning treatments applied to AgND/Ti substrates, including deionized water rinsing, carbonated solution treatment, polarity inversion, and PBS; (b) comparative SERS spectra of R6G (1 × 10^–5^ M) recorded on two identical AgND/Ti substrates with and without PBS conditioning; R6G reference Raman and as-deposited substrate spectra are included for comparison. SEM images of AgNDs grown under the optimized condition (E1, 2.0 V, 60 s) (c) before and (d) after PBS surface conditioning, showing preservation of the hierarchical dendritic morphology. (e) EDS spectrum acquired after PBS conditioning, confirming Ag as the dominant constituent together with contributions from the underlying Ti substrate; (f) SEM image of the region analyzed by EDS, evidencing that the compositional analysis originates from an intact dendritic zone.

Figure 6b compares SERS spectra of R6G acquired on AgND substrates fabricated under identical electrodeposition conditions and measured using identical acquisition parameters, with the only difference being the application of the PBS conditioning step. In the absence of PBS conditioning (red line), the spectrum exhibits additional bands that do not coincide with the characteristic vibrational fingerprint of R6G^76^ (black line), indicating that residual dendrite-associated species contribute to the measured signal even in the presence of an analyte. The major peaks of R6G are listed in Table S1 (Supporting Information). These contributions introduce chemical ambiguity and may lead to erroneous band assignments or artificially enhanced apparent intensities. After PBS conditioning (blue line), these nonanalyte-related features are fully suppressed, yielding a spectrum that closely reproduces the reference R6G fingerprint while preserving the intensity of its characteristic modes. This comparison demonstrates that PBS conditioning enhances chemical selectivity and signal fidelity under practical SERS operating conditions, ensuring that the measured response originates predominantly from the target analyte rather than from substrate-derived residues.

Figure 6c–f evaluates the effect of PBS conditioning on the morphology and composition of the AgND/Ti substrates. SEM images acquired before (Figure 6c) and after PBS conditioning (Figure 6d) show that the hierarchical dendritic architecture remains intact, with no evidence of delamination or structural detachment from the Ti substrate, indicating stable interfacial attachment under the investigated conditions. Elemental analysis of the PBS-treated substrate confirms compositional preservation: the EDS spectrum (Figure 6e) is dominated by Ag signals, with Ti contributions from the underlying metallic substrate, and the conditioning process introduces no additional elemental species. The corresponding SEM image of the analyzed region (Figure 6f) verifies that the EDS signal originates from an intact dendritic area. Overall, these results demonstrate that PBS conditioning preserves both morphology and Ag-rich composition, ensuring that subsequent SERS measurements are not affected by conditioning-induced artifacts.

On the basis of these results, all subsequent SERS measurements reported in this work were performed exclusively on PBS-conditioned AgND/Ti substrates to ensure that the recorded Raman signals originate predominantly from the target analyte, with minimal contribution from substrate-related spectral features. This step is essential for avoiding misinterpretation and for enabling the reliable detection of both small-molecule probes and larger biomolecular species on highly active AgND-based SERS platforms.

### 3.5 Morphology–SERS Correlation and Optimization

To establish a direct relationship between dendritic morphology and SERS performance, the Raman response of Rhodamine 6G (1 × 10^–5^ M) was systematically evaluated for AgNDs grown under the electrochemical and hydrodynamic conditions defined in Section 2.1. R6G was selected as a molecular probe due to its well-defined and widely reported vibrational fingerprint on Ag-based SERS substrates, characterized by prominent bands at approximately 612, 774, 1185, 1311, 1363, 1507, and 1650 cm^–1^,^51^ which are commonly associated with in-plane and out-of-plane aromatic ring vibrations and xanthene/phenyl-related modes [Table S2 (Supporting Information)]. Among these features, the band at 1507 cm^–1^ was selected as the reference peak for quantitative comparison, as it exhibits high intensity and spectral isolation. All substrates were subjected to identical PBS conditioning before analysis to eliminate intrinsic surface-related signals and ensure that the observed Raman response originates exclusively from morphology-dependent electromagnetic enhancement. As is common for dendritic and hotspot-dominated SERS substrates, the reported intensity corresponds to the maximum signal observed among the measured points, reflecting the intrinsic electromagnetic amplification capability of the most active hotspots within each architecture.^49^ Moreover, SERS hotspots are considered to be dendrite branches and are thus localized by optical focusing at the surface.^44^ It should be noted that deposition at 2.0 V in electrolyte E2 was not considered in subsequent analyses, as this condition yields particle-dominated morphologies rather than interconnected AgND architectures; accordingly, all references to 2.0 V discussed hereafter correspond exclusively to electrolyte E1.

Figure 7 summarizes the morphology–SERS correlation by plotting the intensity of the R6G band at 1507 cm^–1^ as a function of deposition time and applied potential under magnetic stirring and static conditions. Under magnetic stirring, a clear nonmonotonic dependence of SERS intensity on both deposition time and applied potential is observed. At short deposition times (30 s), moderate enhancement is obtained across the investigated voltage range, indicating that AgND growth remains underdeveloped. Increasing the deposition time to 60 s results in a pronounced enhancement maximum, with the highest SERS intensity observed at 2.0 V. This condition corresponds to the formation of a highly branched, hierarchically interconnected AgND network, as discussed in Sections 3.2 and 3.3. Further prolonging the deposition time to 120 and 180 s results in a systematic reduction in SERS intensity under convective conditions, despite increased Ag loading. This decrease indicates that continued growth promotes branch thickening, lateral coalescence, and partial loss of fine interbranch junctions, rather than the generation of new high-efficiency hotspots.

**Figure 7:**
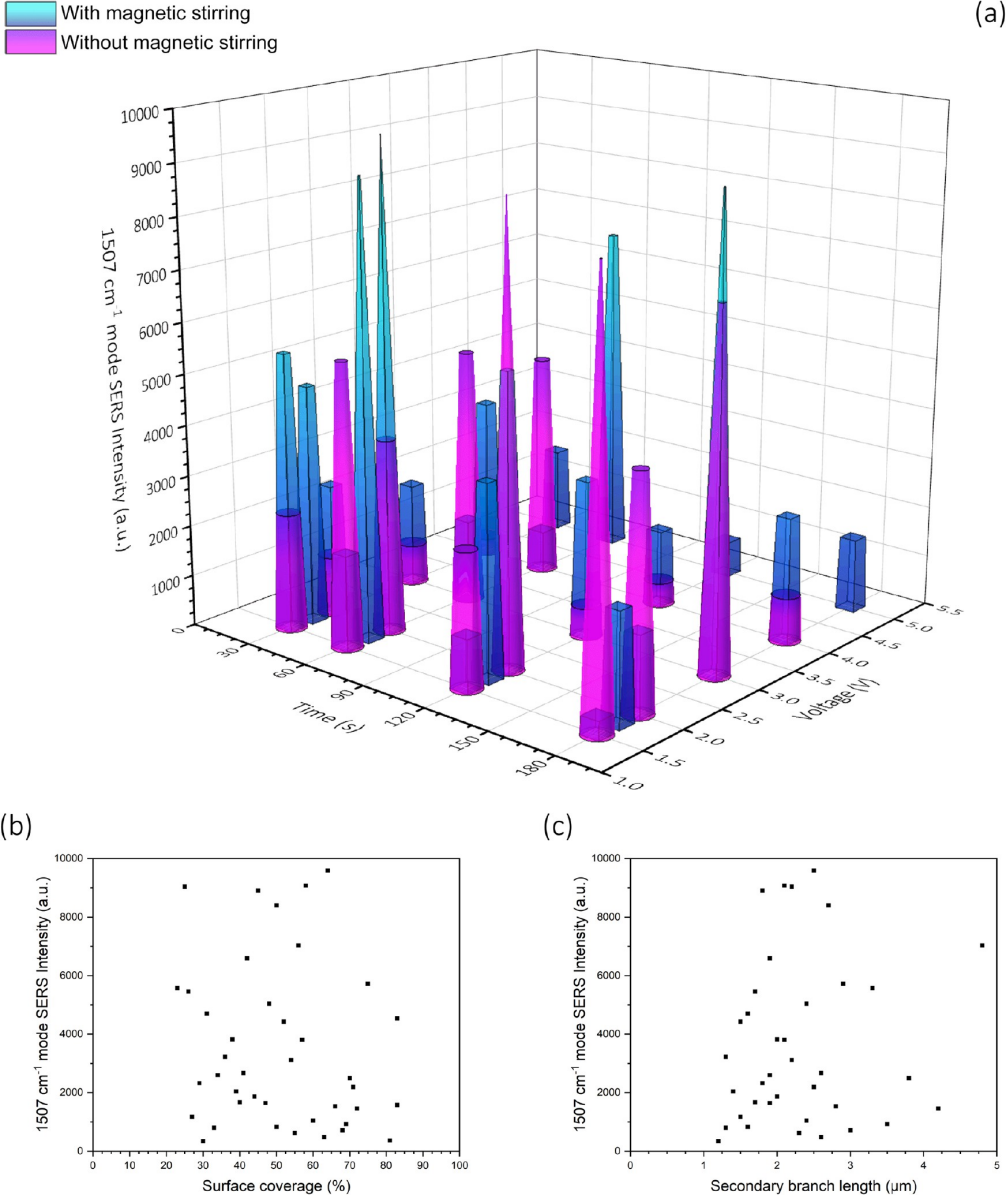
(a) Maximum SERS intensity of the R6G band at 1507 cm^–1^ as a function of deposition time and applied potential for Ag nanodendrites (AgNDs) grown on Ti under magnetic stirring (blue gradient) and without magnetic stirring (purple gradient). The optimal growth condition is identified at 2.0 V for 60 s under convective conditions using electrolyte E1; (b) correlation between SERS intensity and surface coverage, showing a nonmonotonic dependence with maximum enhancement at intermediate coverage levels; (c) correlation between SERS intensity and secondary branch length, indicating that well-developed branching favors enhanced electromagnetic response, although no direct linear relationship is observed.

In contrast, under static conditions, the maximum SERS response is shifted toward longer deposition times. The highest intensity is obtained at 2.0 V for 120 s, indicating that, in the absence of convective transport, extended deposition is required to compensate for diffusion-limited Ag^+^ replenishment and to develop dendritic features capable of sustaining strong electromagnetic enhancement. Although high intensities are also observed at longer times and lower potentials, these responses are associated with increased morphological heterogeneity. The comparison between stirred and static deposition conditions demonstrates that convective mass transport enables the formation of optimal AgND architectures within a narrower growth window. In contrast, diffusion-limited conditions require longer deposition times to approach comparable enhancement levels. Taken together, the results presented in Figure 7 identify AgNDs grown at 2.0 V for 60 s under magnetic stirring as the optimal morphology among all evaluated conditions. This architecture provides the most favorable compromise between dendritic complexity, hotspot density, and structural accessibility, resulting in the highest intrinsic electromagnetic enhancement for R6G. Applied potentials exceeding 2.0 V in electrolyte E1 were not further pursued, as higher electric-field driving forces promote excessive AgND overgrowth, leading to mechanically unstable architectures that readily detach from the Ti substrate during handling and postdeposition processing. Under static conditions, the strongest response is obtained at 2.0 V for 120 s; however, this shift reflects delayed dendritic development under diffusion-limited conditions rather than a fundamentally more efficient hotspot architecture. The complete set of SERS intensity values is provided in Tables S2 and S3 (Supporting Information). A detailed inspection of the morphological parameters summarized in Tables S4 and S5 (Supporting Information), together with the correlations shown in Figure 7b and 7c, reveals that the highest SERS intensities are not associated with the maximum values of individual structural parameters, but rather with an optimal balance between dendritic growth features. Specifically, the most intense Raman responses are consistently observed for AgND architectures exhibiting intermediate surface coverage (∼50–65%) combined with well-developed secondary branching (∼2–3 μm), as clearly evidenced by these correlations. This indicates that SERS enhancement is governed by a regime in which hotspot density is maximized while preserving sufficient interbranch gaps. In contrast, excessive surface coverage (>70%) or prolonged growth leads to branch thickening and structural coalescence, which reduces the availability of effective electromagnetic hotspots despite increased Ag loading. Similarly, insufficient branching or low coverage results in a reduced number of active sites. These findings confirm that SERS performance is dictated by a balanced dendritic architecture rather than by the absolute magnitude of any single morphological parameter. From an experimental standpoint, this optimal regime is achieved through the interplay between deposition time and applied potential, which together control the evolution of dendritic complexity and surface accessibility.

### 3.6 Statistical Mapping Analysis and Reproducibility

In the preceding sections, individual representative substrates were analyzed to establish growth regimes, morphological trends, and optimal deposition windows. Statistical validation and reproducibility were subsequently evaluated exclusively for the optimized condition, as presented below. The spatial uniformity of the optimized AgNDs/Ti substrate (2.0 V, 60 s, magnetic stirring) was evaluated by Raman mapping^83,84^ of the R6G band at 1507 cm^–1^ over a 300 × 200 μm region using 12 measurement points. The resulting intensity map (Figure 8a) shows a nonuniform distribution with localized high-intensity zones, consistent with the hotspot-dominated nature of AgND plasmonic architectures.^85,86,87^ The corresponding optical image (Figure 8b) confirms that the mapped area covers a representative portion of the AgND-coated surface, where the optical contrast and bright features vary across the field of view.

**Figure 8:**
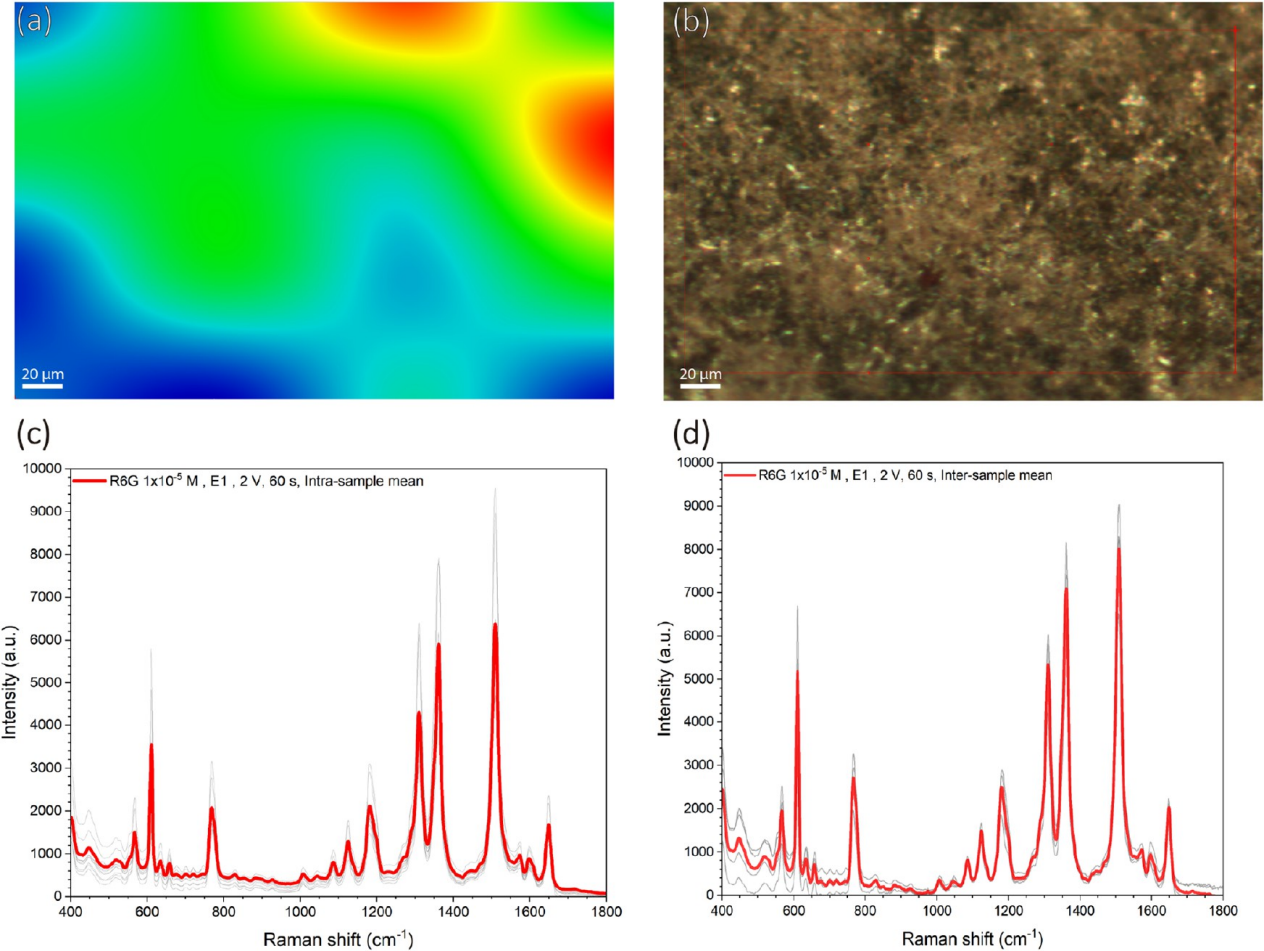
Spatial SERS mapping of the optimized AgNDs (2.0 V, 60 s, magnetic stirring) using the R6G band at 1507 cm^–1^: (a) intensity map over the mapped region, (b) optical image indicating the mapped area, (c) overlaid spectra from the 12 points with the mean spectrum (intrasample), and (d) SERS spectra acquired from four independently prepared substrates, showing high intersample reproducibility (intrasample).

Point-to-point spectral variability (intrasample) is further illustrated by the overlaid spectra (Figure 8c), where the characteristic R6G bands remain consistently detectable across all locations, while their intensities fluctuate. Quantitatively, the 1507 cm^–1^ intensity yields an average of 6386.1 ± 1616.9 au, corresponding to a relative standard deviation (RSD)^52^ of ∼25%. This dispersion reflects the intrinsic heterogeneity of electromagnetic enhancement in AgND networks, where local differences in branch height, junction density, and interbranch gaps produce spatially discrete hotspots within the confocal probing area.^53,88^

The reproducibility of the optimized AgND/Ti substrate was further evaluated by preparing four independent samples (Figure 8d). For each substrate, Raman spectra were acquired under identical instrumental settings, and the mean intensity of the characteristic R6G band at 1507 cm^–1^ was used as the quantitative metric. All four substrates exhibited highly consistent spectral fingerprints, with well-defined R6G bands preserved across the entire spectral window. Minor variations in absolute intensity were observed among samples, reflecting unavoidable differences in dendritic branch density, local junction formation, and nanoscale growth instabilities inherent to potentiostatically grown AgND architectures. Quantitatively, the average SERS intensity across the four substrates was 8173.4 ± 1318.6 au, corresponding to an RSD of approximately 16%. This level of intersample variability demonstrates good fabrication reproducibility for a hotspot-dominated SERS platform and compares favorably with previously reported dendritic and fractal Ag substrates. Importantly, the lower RSD obtained for intersample measurements relative to spatial mapping indicates that the electrodeposition protocol yields consistent average enhancement levels across independently prepared substrates. At the same time, local intensity fluctuations remain governed by the intrinsic spatial distribution of hotspots within each sample. Long-term cycling stability and substrate reusability were not investigated in this work, as the platform is not designed for analyte regeneration or repeated detection cycles. Instead, the focus was placed on fabrication reproducibility and consistent SERS response under controlled conditions. Evaluation of substrate reusability will be addressed in future studies aimed at developing reusable SERS platforms. Taken together, the combined mapping and reproducibility analyses confirm that the optimized AgND/Ti substrates provide a reliable, reproducible SERS response at the substrate level while preserving the high local enhancement required for trace-level detection.

Taken together, the combined mapping and reproducibility analyses confirm that the optimized AgND/Ti substrates provide a reliable, reproducible SERS response at the substrate level while preserving the high local enhancement required for trace-level detection.

### 3.7 Analytical Performance: R6G Concentration-Dependent and Small-Molecule Response

To further evaluate the analytical performance of the optimized substrate, a calibration curve was constructed using the Raman intensity of the R6G band at 1507 cm^–1^ over the concentration range from 1 × 10^–3^ to 1 × 10^–7^ M. As shown in Figure 9a, the semilog plot of intensity versus concentration exhibits a clear linear relationship, indicating a stable analytical response governed by hotspot-mediated enhancement rather than surface saturation effects. Linear regression in this regime yielded the following calibration equation (eq 4):

4 $$I_{LoD}=9704\times \log_{10}\left(C\right)+72883$$

where *I*_*LoD*_ is the Raman intensity (a.u.) and *C* is the analyte concentration (M). Here, 9704 and 72883 represent the slope and intercept of the calibration curve, respectively. The strong linearity observed in this regime confirms that the AgND/Ti substrate enables reliable quantitative analysis within the evaluated concentration range. Following the criterion defined in Section 2.5, the detection threshold was determined using *I*_*LoD*_ = *I*_*ref*_ + 3*SD*_*ref*_. Substitution of this threshold (Table 1) into the calibration equation yields a *LoD* of 3 × 10^–8^ M, demonstrating the high sensitivity of the AgND/Ti platform for trace-level detection.

**Table 1:** Analytical Enhancement Factors (*AEF*) Calculated for 1507 cm^–1^ Raman Peak of Rhodamine 6G (R6G) at Decreasing Concentrations, Using 1 × 10^–3^ M as the Reference (*C*_*ref*_)

R6G concentration (M)	Raman Intensity (a.u.)	*AEF*
1 × 10^–3^ (*C*_*ref*_)	8 (*I*_*ref*_) ± 1 (*SD*_*ref*_)	–
1 × 10^–3^	43540 ± 1235	5 × 10^3^
1 × 10^–4^	38019 ± 4640	4 × 10^4^
1 × 10^–5^	27256 ± 6228	3 × 10^5^
1 × 10^–6^	13714 ± 3095	1 × 10^6^
1 × 10^–7^	4883 ± 2701	6 × 10^6^

**Figure 9:**
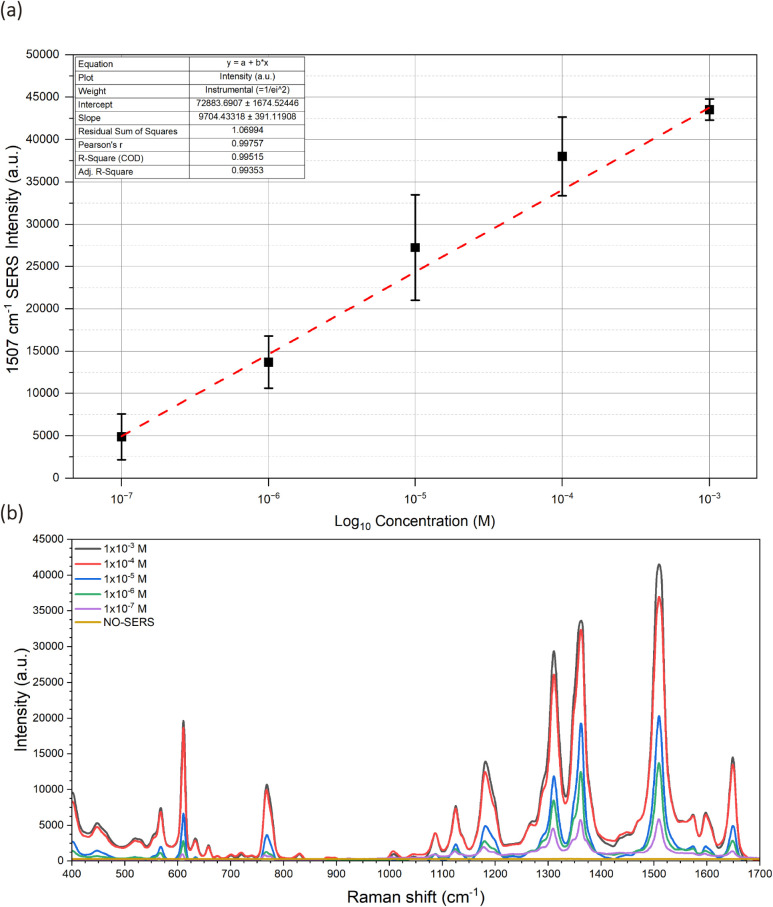
(a) Calibration curve of the SERS intensity of the R6G band at 1507 cm^–1^ as a function of concentration plotted on a semilogarithmic scale; (b) Representative SERS spectra of R6G collected at concentrations from 1 × 10^–3^ to 1 × 10^–7^ M, together with the NO-SERS reference spectrum.

The analytical enhancement factor (*AEF*), calculated according to the procedure described in Section 2.5, increases as the analyte concentration decreases (Table 1). This behavior is characteristic of hotspot-dominated SERS substrates, where electromagnetic enhancement becomes increasingly localized at low surface coverage, confirming the effectiveness of the AgND architecture for ultratrace detection.

To evaluate the versatility of the optimized AgND/Ti substrate, additional SERS measurements were performed using methylene blue (MB) and crystal violet (CV) as representative aromatic dyes with distinct molecular structures. Figure S2a shows the SERS spectrum of MB (1 × 10^–5^ M),^48^ where the characteristic bands at ∼885, 949, 1036, 1180, 1396, 1501, and 1620 cm^–1^ are clearly resolved, in good agreement with previously reported assignments for MB on Ag-based substrates. Similarly, the SERS spectrum of CV at 1 × 10^–5^ M^89^ [Figure S2b] exhibits intense and well-defined bands at ∼800, 915, 1170, 1360, 1380, 1580, and 1615 cm^–1^, corresponding to ring stretching and C–N vibrational modes of the triphenylmethane framework. The clear detection of both MB and CV confirms that the AgND architecture provides robust electromagnetic enhancement for chemically distinct small-molecule probes, demonstrating that the optimized substrate is not limited to a single reporter molecule.

To place the performance of the AgND/Ti substrate developed in this work in context, Table 2 provides a comparative overview of representative SERS platforms reported for molecular dye detection. The surveyed studies include substrates fabricated via DC magnetron sputtering, thermal evaporation, sol–gel processing, and UV-assisted deposition. While these approaches can deliver competitive analytical enhancement factors and low detection limits, they often rely on vacuum-based systems or multistep chemical routes, which increase fabrication complexity and constrain scalability. The comparison considers key parameters, including substrate architecture, fabrication strategy, target analyte, *AEF*, and *LoD*. Beyond sensitivity alone, scalability and practical implementation are critical for real-world SERS applications. In this regard, the PED strategy employed here offers clear advantages, as it is low-cost, readily scalable to large-area substrates, and avoids the need for vacuum infrastructure or complex processing steps. Although some vacuum-based or chemically intensive methods report lower *LoD* values, the present AgND/Ti platform offers a favorable balance of performance, simplicity, and scalability, making it well-suited for practical monitoring applications. Notably, to the best of our knowledge, no prior reports have described the direct potentiostatic growth of AgNDs on nonanodized metallic Ti substrates for SERS applications. This distinguishes the present approach from previous Ag–Ti systems that rely on oxide nanostructures, template-assisted growth, or indirect deposition routes, enabling direct electrical contact, reduced fabrication variability, and simplified integration. Regarding stability, it is well recognized that Ag nanostructures—particularly highly branched dendritic architectures—are susceptible to oxidation and morphological evolution, which can affect long-term signal reproducibility. Although extended durability tests were beyond the scope of this study, the results demonstrate that the AgND/Ti substrates deliver high sensitivity and reproducible performance, while identifying stability optimization as an important direction for future work.

**Table 2:** Comparative Performance of AgND/Ti Nanostructured SERS Substrates^a^

Substrate Composition	Synthesis Method	Analyte	*AEF*	*LoD*	Ref.
AgNP/TiO_2_ Nanotubes	Thermal Evaporation/Constant Voltage Anodization	MB	2 × 10^5^	1 × 10^–8^	^90^
Ag@mTiO_2_ NPs	Hydrothermal Process/Chemical Method	MB	–	1 × 10^–8^	^91^
AgNPs/TiO_2_	Ultraviolet Light-Induced/Sol–Gel	R6G	–	1 × 10^–8^	^92^
AgNP/TiO_2_ Nanotubes	DC Magnetron Sputtering/Arc Ion Plating Method	R6G	1 × 10^9^	1 × 10^–8^	^93^
AgNDs/Ti	Potentiostatic Electrodeposition	R6G	6 × 10^6^	3 × 10^–8^	this work
AgNP/TiO_2_ Nanosheet	DC Magnetron Sputtering/Hydrothermal Method	R6G	1 × 10^5^	1 × 10^–9^	^94^
AgTNP@TiO_2_@Ag core–satellite	Chemistry Method	MB	–	1 × 10^–10^	^95^
AgNP/TiO_2_ Nanotubes and Nanograss	DC Magnetron Sputtering/Constant Voltage Anodization	MB	1 × 10^5^	1 × 10^–12^	^96^
AgNP/TiO_2_ Nanorods	Chemistry Method	MG	4 × 10^5^	1 × 10^–12^	^97^
AgNP/TiO_2_ Nanospheres	Ultraviolet Light-Induced/Commercial Nanospheres	R6G	7 × 10^10^	1 × 10^–12^	^98^
AgNP/TiO_2_ Nanotubes	DC Magnetron Sputtering/Constant Voltage Anodization	R6G	–	1 × 10^–14^	^99^

a R6G: rhodamine 6G; MB: methylene blue; MG: malachite green.

### 3.8 Compatibility with Biomolecular Detection

While the analytical performance of AgND/Ti substrates has been established using small-molecule Raman reporters, their practical relevance for biosensing applications ultimately depends on their ability to interact with larger, chemically complex biomolecules. In contrast to small dyes, biomolecular species such as proteins, antibodies, and antigens present additional challenges for SERS detection due to their size, conformational flexibility, and limited accessibility to highly confined nanoscale hotspots. Therefore, evaluating whether the optimized AgND architecture can support both direct biomolecular detection and stable surface functionalization is essential to assess its versatility beyond model analytes. Representative biomolecules were deliberately selected to probe different levels of structural and chemical complexity. BSA was employed as a model globular protein to validate backbone-related SERS signatures,^100–102,103^ while gliadin was chosen as a cysteine-rich protein to assess sensitivity to disulfide-linked motifs.^104,105,106^ The AFP antibody–antigen system was selected as a well-established biosensing model to evaluate surface functionalization compatibility and antibody-mediated interfaces,^75^ rather than for clinical quantification. In this section, the compatibility of the optimized substrates with biomolecular sensing is examined through direct SERS detection, molecular linker optimization, and antibody immobilization via MPA–EDC/NHS chemistry.^51^

Figure 10 illustrates the direct SERS detection of structurally diverse biomolecular species on the optimized AgND/Ti substrates, including BSA (a), Ab-Gli (b), Ab-AFP (c), and AFP (d). Despite their chemical and structural complexity, all biomolecules exhibit reproducible SERS responses dominated by vibrational regions characteristic of protein backbones, confirming that the recorded signals originate predominantly from the biomolecular species.

**Figure 10:**
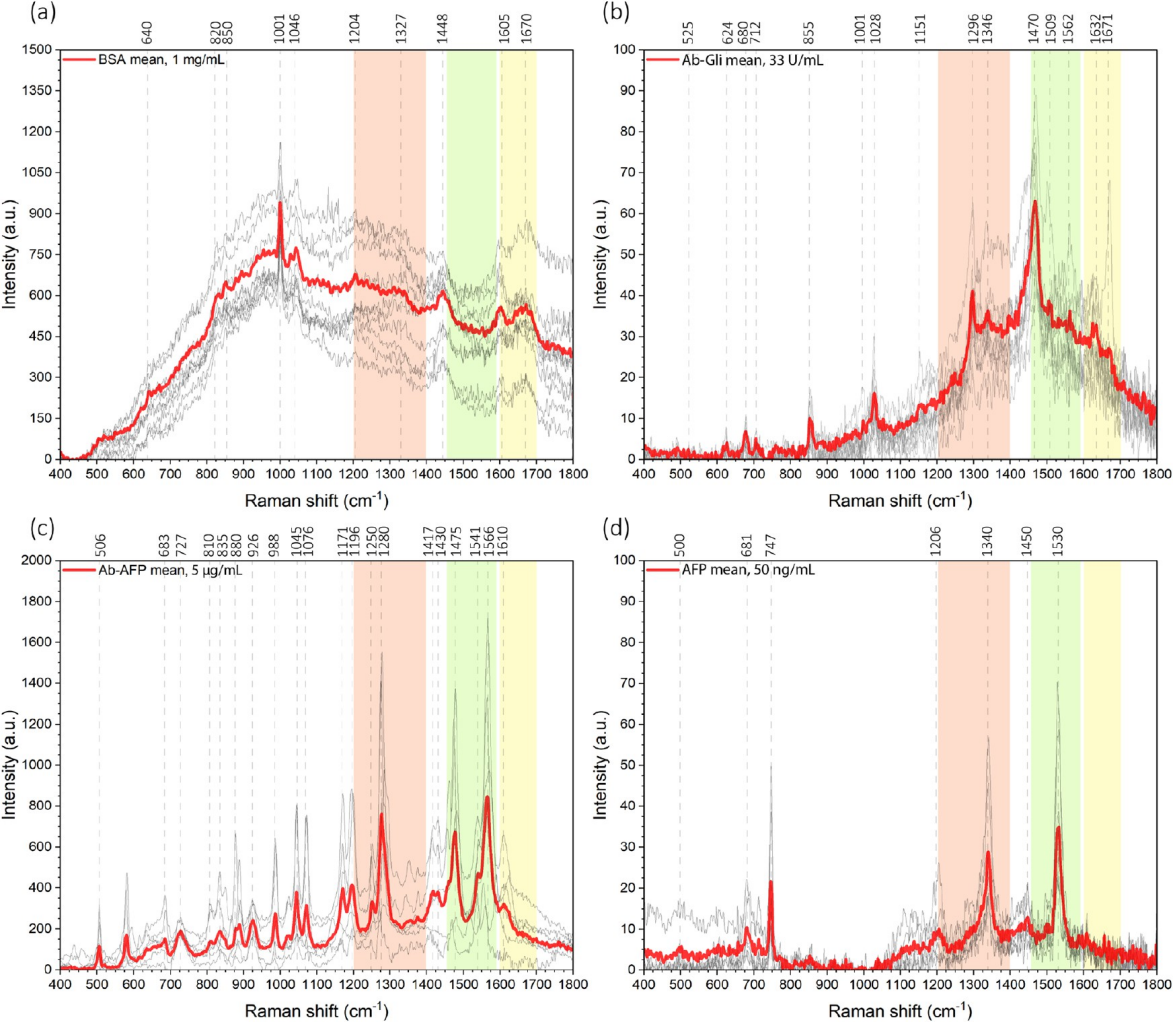
SERS spectra of biomolecular species directly measured on the optimized AgND substrates: (a) bovine serum albumin (BSA, 1 mg/mL), (b) Ab-Gli (33 U/mL), (c) monoclonal anti-α-fetoprotein antibody (Ab-AFP, 50 μg/mL), and (d) α-fetoprotein antigen (AFP, 50 ng/mL). Shaded regions highlight the characteristic Amide III (1200–1400 cm^–1^, red), Amide II (1480–1600 cm^–1^, green), and Amide I (1600–1680 cm^–1^, yellow) bands, together with additional contributions from C–C, C–N, and C–H vibrational modes. Gray curves correspond to individual spectra, while the red trace represents the mean response.

Across all spectra, three broad and consistent vibrational regions can be identified and assigned to the Amide III (1200–1400 cm^–1^, red zone), Amide II (1480–1600 cm^–1^, green zone), and Amide I (1600–1680 cm^–1^, yellow zone) bands. These regions arise from coupled C–N stretching, N–H bending, and C=O stretching modes of the peptide backbone and are commonly used as spectroscopic signatures of proteinaceous materials.^107,108,109^ Their simultaneous presence across all four biomolecular systems provides strong evidence that the AgND substrates can couple plasmonic enhancement to large macromolecular structures without the need for labeling or surface functionalization.^14,42^ Based on established Raman and SERS studies of proteins and amino acids (Table S6, Supporting Information),^110,111^ a more detailed assignment of the characteristic Raman bands associated with the analyzed biomolecular species is presented in Table S7–S10 (Supporting Information). In these tables, the main vibrational features are tentatively correlated with specific amino acid residues and protein backbone modes for BSA, Ab-Gli, Ab-AFP, and AFP. These assignments were restricted to medium-to-very-strong Raman-active modes (m, s, and vs), thereby minimizing contributions from weak and poorly resolved bands. This approach supports the interpretation of the amide-dominated spectral regions and reinforces the biomolecular origin of the detected signals, without implying unambiguous identification of individual residues.

Compared with small-molecule Raman reporters^14^ such as R6G, MB, or CV, the biomolecular spectra exhibit a notably greater intensity dispersion, as reflected in the broader distribution of individual spectra around the mean response.^112^ This behavior is intrinsically linked to the large molecular size, conformational flexibility, and steric complexity of proteins and antibodies.^109^ Unlike planar dye molecules, which can readily penetrate narrow nanogaps and uniformly sample highly confined hotspots, biomolecules interact with the plasmonic surface through partial contact and orientation-dependent adsorption.^113,114^ As a result, only specific domains of the macromolecule may reside within regions of maximum electromagnetic enhancement, leading to spatially heterogeneous but reproducible SERS responses.^115^ This effect is a well-known characteristic of direct biomolecular SERS and does not indicate instability of the substrate but rather reflects the geometric and structural constraints imposed by large analytes.

Notably, in the spectra of Ab-Gli and Ab-AFP, additional low-frequency contributions are observed in regions associated with sulfur-containing bonds. In particular, bands attributable to S–S stretching modes (500–550 cm^–1^) are consistent with the presence of disulfide bridges, which play a fundamental structural role in stabilizing antibody domains and cysteine-rich proteins such as gliadin.^116,117,118^ The observation of these features further supports the direct biomolecular origin of the detected signals. It demonstrates that the AgND architecture can enhance vibrational modes associated with chemically specific structural motifs within complex macromolecules.^119^

Despite differences in molecular size, composition, and structural organization, all biomolecules yield clear SERS signatures within the same amide-dominated spectral region. This observation highlights a key advantage of the AgND architecture: its open, multiscale morphology provides accessible plasmonic field regions that extend beyond highly confined nanogaps, enabling interaction with large biomolecules that would otherwise be excluded from classical hotspot geometries. Consequently, the AgND/Ti substrates support direct SERS detection across a wide range of molecular sizes, from small dyes to structurally complex proteins and antibodies.

Figure 11 summarizes the optimization of the molecular linker layer and the subsequent antibody immobilization strategy used to enable controlled bioconjugation on the AgND/Ti substrates. As a thiolated short-chain linker, MPA binds to Ag through strong Ag–S interactions while exposing terminal carboxyl groups suitable for downstream EDC/NHS coupling.^51,75^ Figure 11a compares the SERS signatures of MPA deposited at concentrations of 1 × 10^–2^, 1 × 10^–3^, and 1 × 10^–4^ M, highlighting characteristic conformational bands assigned to gauche (653 cm^–1^) and trans/anti (735 cm^–1^) chain arrangements, together with a prominent band associated with the terminal carboxyl functionality (926 cm^–1^).^120^ A more detailed assignment of the MPA-related vibrational modes is provided in Table S11 (Supporting Information). Among the evaluated conditions, the 1 × 10^–3^ M layer yields the highest overall intensity, indicating an optimal balance between surface coverage and plasmonic coupling. At higher concentrations (1 × 10^–2^ M), the reduced signal intensity is consistent with the formation of an overly dense organic layer that increases the effective spacing between the enhanced electromagnetic field regions and the vibrational reporters, partially attenuating hotspot-mediated enhancement.

**Figure 11:**
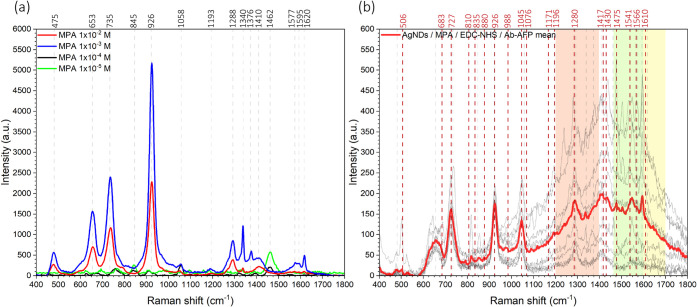
(a) SERS spectra of MPA deposited at 1 × 10^–2^, 1 × 10^–3^, and 1 × 10^–4^ M on optimized AgNDs, highlighting gauche/trans (anti) conformational features and the terminal carboxyl band. (b) SERS spectrum after sequential functionalization with MPA (1 × 10^–3^ M), EDC/NHS activation, and Ab-AFP immobilization. Red dashed lines indicate characteristic Ab-AFP bands, while gray dashed lines correspond to MPA-related vibrational features, allowing visualization of the overlapping spectral contributions from the linker layer and the immobilized protein.

In contrast, at lower concentrations (1 × 10^–4^ M), the diminished response suggests insufficient linker coverage relative to the large effective surface area of the AgND architecture. The relative intensities of the gauche and trans/anti modes further support an orientation- and packing-dependent response governed by SERS surface-selection rules, in which vibrational modes are differentially enhanced depending on both the local electromagnetic field distribution within AgND hotspots and the orientation of the linker molecules at the Ag interface.^121,122^ Based on these results, a concentration of 1 × 10^–3^ M MPA was selected as the optimized functionalization condition for subsequent bioconjugation.

Figure 11b shows the SERS response of the fully functionalized substrate after sequential treatment with MPA, EDC/NHS activation, and Ab-AFP immobilization. After coupling, the spectrum simultaneously retains identifiable MPA-related features, including residual gauche and trans/anti conformational signatures, while exhibiting clear protein-associated vibrational regions corresponding to the amide bands. To facilitate spectral interpretation, Figure 11b also includes dashed reference lines corresponding to the characteristic bands of Ab-AFP (red dashed lines) and MPA (gray dashed lines), enabling a clear visualization of the overlapping contributions from the linker layer and the immobilized protein. This coexistence indicates that the linker layer remains present while enabling the covalent attachment of the biomolecular species. A decrease in overall SERS intensity is observed after functionalization compared with the MPA-only condition. This attenuation is expected and can be attributed to the increased distance between the plasmonic hotspots and the vibrational reporters, as well as to partial surface coverage by the organic linker layer and immobilized biomolecules. In SERS systems, such distance-dependent effects are well-known to reduce enhancement efficiency without necessarily indicating degradation of the underlying plasmonic response. Notably, the band associated with the terminal carboxyl group shows reduced relative intensity compared to the MPA-only condition, consistent with the chemical activation of carboxyl groups into NHS esters and their subsequent conversion into amide bonds upon reaction with antibody amines.^123,124^ Despite the observed attenuation, the presence of well-defined amide-related spectral features demonstrates that the substrate remains SERS-active after functionalization and is capable of supporting biomolecular detection. Although complementary surface chemical analysis (e.g., XPS) could provide direct information about the oxidation state of Ag after functionalization, the present study evaluates substrate performance based on the retention of a functional SERS response under the applied experimental conditions.

Collectively, these spectral changes provide strong spectroscopic evidence that the AgND substrate supports controlled surface functionalization and stable covalent antibody immobilization, establishing a functional interface suitable for biosensing-oriented SERS configurations.

## 4 Conclusion

This work demonstrates that nonanodized metallic Ti provides an effective, experimentally simple platform for the PED of AgNDs, with dendritic morphology strongly influenced by electrolyte composition and hydrodynamic conditions. The presence of a supporting electrolyte and magnetic stirring enables sustained tip-driven growth behavior, leading to the formation of an optimal SERS-active AgND architecture at 2.0 V for 60 s. This optimal performance is associated with an experimentally observed balanced dendritic morphology characterized by intermediate surface coverage and well-developed secondary branching, which together maximize hotspot density while avoiding excessive structural coalescence. Postdeposition surface conditioning plays a critical role, as mild PBS treatment effectively suppresses intrinsic background signals while preserving dendritic morphology and establishing a chemically compatible interface for subsequent molecular and biomolecular interactions.

The optimized AgND/Ti substrates exhibit robust analytical performance, achieving a *LoD* of 3 × 10^–8^ M for R6G and consistent enhancement for chemically distinct dyes (MB and CV). Importantly, the same substrates are compatible with direct biomolecular SERS detection and controlled surface functionalization, enabling MPA–EDC/NHS-mediated antibody immobilization while maintaining strong and reproducible SERS response. These results highlight the capability of the platform to operate as a protein-compatible and biofunctional SERS interface.

Despite these promising results, several limitations should be acknowledged. The present study does not include detailed electrochemical kinetic analysis (e.g., cyclic voltammetry or chronoamperometry), nor does it address substrate reusability or cycling stability, as the platform was not designed for analyte regeneration. In addition, complementary surface chemical characterization techniques, such as XPS or FTIR, were not employed, and the analytical performance was evaluated under controlled model conditions without matrix-based validation. These aspects define the current scope of the work, which is focused on establishing morphology–performance relationships and demonstrating spectroscopic functionality under well-defined conditions. Future work will focus on addressing these limitations by incorporating electrochemical mechanistic studies, evaluating substrate reusability, implementing advanced surface characterization, and extending the platform toward real-sample analysis.

Overall, electrolyte- and hydrodynamics-controlled growth provides a reliable and scalable route for the fabrication of AgNDs on metallic Ti, offering a versatile foundation for the development of advanced SERS-based molecular and biomolecular detection platforms.

## Supplementary Material



## Data Availability

The original contributions presented in this study are included in the article. Further inquiries can be directed to the corresponding authors.

## Notes

As-deposited AgNDs on Ti prior to any postdeposition cleaning; major peaks for Rhodamine 6G; SERS intensities at 1507 cm^–1^ for AgNDs grown under magnetic stirring and without magnetic stirring; main morphological parameters of AgNDs grown under magnetic stirring and without magnetic stirring conditions; SERS spectra of (a) methylene blue (MB, 1 × 10^–5^ M) and (b) crystal violet (CV, 1 × 10^–5^ M); major protein-related Raman bands for BSA, Ab-Gli, Ab-AFP, and AFP (PDF)
