# Supplementary material for: Electrolyte- and Hydrodynamics-Controlled Potentiostatic Growth of Ag Nanodendrites on Metallic Ti for SERS Detection
Source: ACS Omega. 2026 May 29;11(22):32691–712. doi: 10.1021/acsomega.6c01538 (PMC13261586; doi:10.1021/acsomega.6c01538)
Supplement: Supplementary file 1 [file ao6c01538_si_001.pdf]

Electrolyte- and Hydrodynamics-Controlled Potentiostatic Growth of Ag Nanodendrites on Metallic Ti for SERS Detection

Marcos Luna-Cervantes[1*], Erick Octavio Santos-Santiago[1], Diana Jiménez-Girón[1], José Luis Zamora-Navarro[1], Antonio de Jesús García-Chávez[2], Yuri Okolodkov[3], Jorge Bertín Santaella-González[4], Julián Hernández-Torres[1], Irma Yadira Izaguirre-Hernández[5], Pablo Thomas-Dupont[6], Luis Zamora-Peredo[1*]

[1]Centro de Investigación en Micro y Nanotecnología, Universidad Veracruzana, Av. Adolfo Ruiz Cortines 455, col. Costa Verde, 94294, Boca del Río, México.
[2]Doctorado en Ciencias e Ingeniería, Universidad Autónoma de Baja California, Carretera Transpeninsular Ensenada - Tijuana 3917, col. Playitas, 22860, Ensenada, México
[3]Instituto de Ciencias Marinas y Pesquería, Universidad Veracruzana, Miguel Hidalgo 617, Río Jamapa, 94290, Boca del Río, México.
[4]Facultad de Ciencias Químicas / Facultad de Ingeniería Mecánica y Ciencias Navales, Universidad Veracruzana, Av. Adolfo Ruiz Cortines 455, col. Costa Verde, 94294, Boca del Río, México.
[5]Facultad de Bioanálisis, Universidad Veracruzana, Iturbide s/n Esquina Carmen Serdán, 91700, Veracruz, México.
[6]Instituto de Investigaciones Médico Biológicas, Universidad Veracruzana, Iturbide s/n entre Carmen Serdán y 20 de Noviembre, 91700, Veracruz, México

[*]e-mail: marcoslc@ens.cnyn.unam.mx, luiszamora@uv.mx

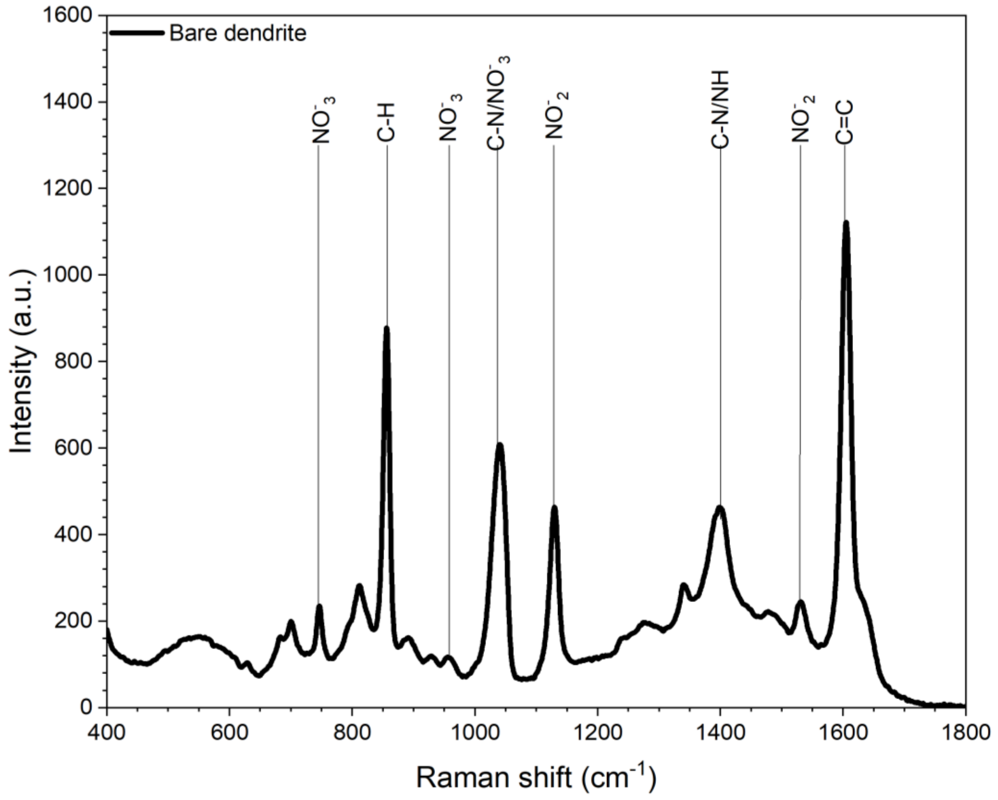

Figure S1. Raman spectrum of as-deposited Ag nanodendrites on Ti prior to any post-deposition cleaning, showing intense background features originating from surface-bound residues and synthesis-related contaminants.

Table S1. Assignments of major peaks for Rhodamine 6G (R6G) [76].

Raman Shift (cm^{-1})	Band assignments
612	Ring in-plane bending in xanthene/phenyl rings C-C
770	Out-of-plane C–H bending
1087	In-plane C–H bending coupled with ring vibration
1127	In-plane C–H bending
1183	In-plane C–H bending (xanthene ring)
1310	Mixed ring stretching with contribution from amino substituent C–C + C–N
1362	Ring stretching vibration of the xanthene framework C-C
1444	Stretching vibration of the amino group C-N
1507	Symmetric aromatic ring stretching (xanthene) C-C
1573	Aromatic ring stretching (phenyl/xanthene) C-C
1596	High-frequency aromatic ring stretching C-C
1648	Strong aromatic ring stretching (xanthene framework) C-C

Table S2. Maximum SERS intensities at 1507 cm^{-1} for Ag nanodendrites grown under magnetic stirring.

Time (s)	Electrolyte	Voltage (V)	1507 cm^{-1} mode SERS Intensity (a.u.)
30	E1	1.50	5457
30	E1	1.75	4702
30	E1	2.00	2601
60	E1	1.50	1870
60	E1	1.75	8907
60	E1	2.00	9592
120	E1	1.50	1048
120	E1	1.75	3806
120	E1	2.00	5723
180	E1	1.50	364
180	E1	1.75	2191
180	E1	2.00	1580
30	E2	3.00	2042
30	E2	4.00	3227
30	E2	5.00	1674
60	E2	3.00	1645
60	E2	4.00	833
60	E2	5.00	6592
120	E2	3.00	3117
120	E2	4.00	1534
120	E2	5.00	712
180	E2	3.00	9077
180	E2	4.00	2495
180	E2	5.00	1459

Table S3. Maximum SERS intensities at 1507 cm^{-1} for Ag nanodendrites grown without magnetic stirring.

Time (s)	Electrolyte	Voltage (V)	1507 cm^{-1} mode SERS Intensity (a.u.)
30	E1	1.50	2323
30	E1	2.00	1174
60	E1	1.50	5577
60	E1	2.00	3820
120	E1	1.50	2671
120	E1	2.00	9040
180	E1	1.50	8404
180	E1	2.00	4538
30	E2	3.00	800
30	E2	4.00	345
60	E2	3.00	5039
60	E2	4.00	4428
120	E2	3.00	619
120	E2	4.00	483
180	E2	3.00	7031
180	E2	4.00	925

Table S4. Morphological parameters of Ag nanodendrites, including primary trunk and secondary branch lengths, and surface coverage, grown under magnetic stirring.

Time (s)	Electrolyte	Voltage (V)	Primary trunk length (μm)	Secondary branch length (μm)	Surface coverage (%)
30	E1	1.50	8.8 ± 2.1	1.7 ± 0.5	26
30	E1	1.75	12.6 ± 2.2	1.3 ± 0.2	28
30	E1	2.00	11.3 ± 1.9	1.9 ± 0.7	46
60	E1	1.50	13.7 ± 3.7	1.3 ± 0.3	36
60	E1	1.75	14.5 ± 3.1	1.8 ± 0.2	45
60	E1	2.00	14.8 ± 3.3	2.5 ± 0.5	64
120	E1	1.50	12. ± 2.9	1.1 ± 0.2	49
120	E1	1.75	15.1 ± 1.3	1.4 ± 0.2	56
120	E1	2.00	15.6 ± 2.5	2.9 ± 1.1	75
180	E1	1.50	13.0 ± 4.0	1.5 ± 0.5	55
180	E1	1.75	19.4 ± 4.4	1.5 ± 0.4	71
180	E1	2.00	N/A	N/A	83
30	E2	3.00	2.8 ± 0.3	0.6 ± 0.2	19
30	E2	4.00	3.1 ± 0.5	0.6 ± 0.2	23
30	E2	5.00	14.6 ± 1.8	2.0 ± 0.3	28
60	E2	3.00	12.5 ± 4.2	1.9 ± 0.5	23
60	E2	4.00	8.9 ± 2.4	2.5 ± 0.4	29
60	E2	5.00	19.3 ± 3.7	1.9 ± 0.5	42
120	E2	3.00	15.2 ± 3.5	1.4 ± 0.3	36
120	E2	4.00	24.9 ± 5.3	3.1 ± 1.0	46
120	E2	5.00	22.3 ± 3.8	2.3 ± 0.5	58
180	E2	3.00	18.3 ± 4.8	2.1 ± 0.4	58
180	E2	4.00	23.7 ± 3.4	4.0 ± 1.2	70
180	E2	5.00	24.6 ± 7.0	3.0 ± 1.0	68

N/A: Not reliably measurable due to structural overlap and indistinguishable dendritic features.

Table S5. Morphological parameters of Ag nanodendrites, including primary trunk and secondary branch lengths, and surface coverage, grown without magnetic stirring.

Time (s)	Electrolyte	Voltage (V)	Primary trunk length (μm)	Secondary branch length (μm)	Surface coverage (%)
30	E1	1.50	4.7 ± 0.7	N/A	20
30	E1	2.00	5.4 ± 1.0	1.5 ± 0.3	23
60	E1	1.50	11.5 ± 3.0	3.3 ± 0.2	23
60	E1	2.00	7.2 ± 1.1	1.9 ± 0.4	26
120	E1	1.50	12.6 ± 1.5	3.0 ± 0.9	43
120	E1	2.00	7.9 ± 1.2	2.2 ± 0.6	25
180	E1	1.50	11.6 ± 1.2	2.7 ± 0.7	50
180	E1	2.00	11.9 ± 2.2	2.3 ± 0.4	38
30	E2	3.00	4.8 ± 1.0	2.1 ± 0.4	22
30	E2	4.00	12.5 ± 2.0	2.5 ± 0.6	30
60	E2	3.00	11.1 ± 3.8	2.9 ± 0.7	26
60	E2	4.00	19.6 ± 3.1	2.6 ± 0.7	38
120	E2	3.00	15.9 ± 1.7	2.9 ± 0.7	36
120	E2	4.00	16.6 ± 2.7	1.7 ± 0.1	60
180	E2	3.00	21.4 ± 5.2	4.8 ± 1.2	56
180	E2	4.00	17.2 ± 2.8	2.1 ± 1.0	72

N/A: Not reliably measurable due to indistinguishable dendritic features.

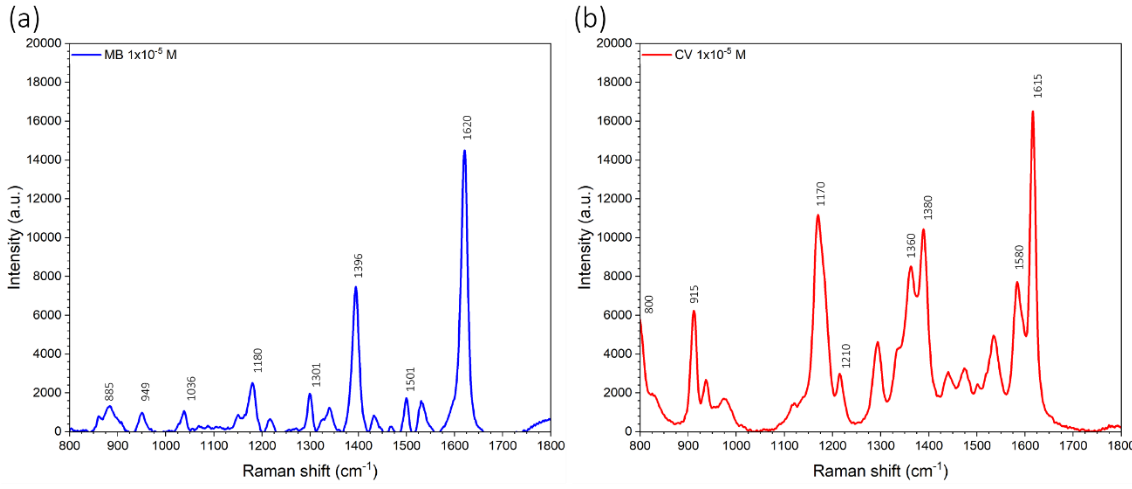

Figure S2. SERS spectra of (a) methylene blue (MB, 1×10^{-5} M) and (b) crystal violet (CV, 1×10^{-5} M) acquired on the optimized Ag nanodendritic substrate, showing their characteristic vibrational fingerprints.

Table S6. Raman bands of amino acids by spectral region [110] [111].

Amino acid	Abbrev.	300–400	400–500	500–600	600–700	700–800	800–900	900–1000	1000–1100	1100–1200	1200–1300	1300–1400	1400–1500	1500–1600	1600–1700
Alanine	Ala	399	—	533	653	771	852	922	1021	1115<br>1149	1239	1308<br>1361<br>1378	1411<br>1464<br>1485	1500<br>1599	—
Arginine	Arg	—	—	—	611<br>652	798	873	926<br>985	1036<br>1083	1100<br>1123<br>1199	1264	1310<br>1331<br>1364	1423<br>1443<br>1477	—	—
Aspartic acid	Asp	—	—	—	660	749<br>779	873	902<br>939<br>992	1084	1121<br>1141	1251<br>1262	1338<br>1362	1409<br>1426	1507	1695
Cystine	Cys	—	455<br>499	542	613<br>678	785	844<br>873	967	1041<br>1092	1134<br>1196	—	1301<br>1341<br>1385	1410<br>1488	—	1625
Glutamic acid	Glu	—	—	—	623<br>669	750<br>781	873	917<br>988	1008<br>1042<br>1080	1164<br>1182	1275<br>1287	1319<br>1346<br>1379	1422<br>1462	—	1637<br>1682
Glycine	Gly	359	496	—	603<br>697	—	894	—	1036	1107<br>1143	—	1327	1413<br>1442<br>1458	1515<br>1568	—
Histidine	His	—	—	539	626<br>657<br>680	731<br>784	806<br>825<br>854	919<br>929<br>964<br>977	1062<br>1088	1113<br>1141<br>1176	1225<br>1252<br>1272	1319<br>1336<br>1349	1409<br>1431<br>1475	1500<br>1573	—
Isoleucine	Ile	—	—	536<br>557	675	710<br>749<br>769	800<br>825<br>852<br>873	918<br>964<br>993	1017<br>1033<br>1089	1134<br>1168<br>1191	1257<br>1273	1310<br>1329<br>1355<br>1398	1421<br>1450	1514<br>1583	1619
Leucine	Leu	—	—	—	670	771	836<br>848	924<br>948<br>966	1028<br>1083	1131<br>1178<br>1187	1243<br>1296	1315<br>1342<br>1370	1410<br>1457	1514<br>1583	1623
Lysine	Lys	—	—	—	625	741<br>785	877	912<br>946<br>975<br>988	1036<br>1055<br>1072<br>1097	1141<br>1168	1205<br>1226<br>1257<br>1283	1305<br>1341<br>1361<br>1399	1433<br>1456<br>1464<br>1485	1570	1609
Methionine	Met	—	—	—	645<br>659<br>682	721<br>765	805<br>877	923<br>953<br>984	1040<br>1075	1122<br>1152<br>1175	1245<br>1265<br>1280	1321<br>1335<br>1355	1415<br>1447	1511	1621
Phenylalanine	Phe	—	469	525	606<br>622<br>684	748<br>786	821<br>835<br>852	915<br>954	1005<br>1036	1130<br>1158<br>1188	1214<br>1293	1310<br>1343	1413<br>1438<br>1448	1589	1608

Proline	Pro	—	—	—	642 (m)	—	843 (m)<br>899 (m)	921 (m)<br>952 (w)<br>986 (w)	1035 (w)<br>1055 (vw)<br>1084 (m)	1173 (w)	1239 (m)<br>1266 (vw)<br>1290 (vw)	1376 (vw)	1453 (vw)<br>1477 (vw)	1548 (vw)	—
Serine	Ser	—	—	—	610 (m)	—	805 (m)<br>814 (m)<br>854 (s)	922 (w)<br>969 (m)	1010 (s)	1127 (m)	1220 (m)	1301 (m)<br>1327 (s)	1417 (m)<br>1464 (m)	—	1630 (w)
Threonine	Thr	—	418 (w)<br>447 (m)<br>488 (w)	564 (s)	—	702 (w)<br>742 (w)<br>774 (w)	872 (s)	904 (w)<br>932 (m)	1031 (w)<br>1045 (w)	1116 (s)<br>1195 (w)	1251 (w)	1341 (s)	1419 (m)<br>1467 (w)<br>1483 (w)	1548 (w)	1600 (vw)<br>1621 (vw)<br>1651 (vw)
Tryptophan	Trp	—	—	—	627 (w)<br>685 (vw)	707 (w)<br>740 (w)<br>756 (s)<br>779 (w)	805 (w)<br>849 (w)<br>875 (m)	928 (vw)<br>961 (vw)<br>988 (vw)	1010 (s)<br>1068 (vw)<br>1078 (vw)	1105 (vw)<br>1120 (vw)	1233 (m)<br>1253 (w)<br>1293 (vw)	1315 (vw)<br>1340 (w)<br>1360 (m)	1426 (m)<br>1460 (w)<br>1489 (w)	1559 (m)<br>1579 (w)	1619 (w)
Tyrosine	Tyr	—	—	—	641 (m)	715 (w)<br>742 (vw)<br>798 (m)	830 (s)<br>847 (m)<br>881 (vw)<br>897 (vw)	986 (m)	1044 (m)<br>1099 (vw)	1115 (vw)<br>1156 (w)<br>1180 (m)	1201 (m)<br>1215 (w)<br>1248 (w)<br>1267 (w)<br>1285 (vw)	1328 (m)<br>1366 (w)	1436 (w)	—	1615 (m)
Valine	Val	—	430 (w)	542 (s)	665 (m)	715 (w)<br>753 (m)<br>776 (s)	825 (m)<br>850 (s)	902 (w)<br>948 (s)<br>965 (m)	1035 (w)<br>1066 (m)	1126 (m)<br>1144 (w)<br>1179 (vw)<br>1192 (m)	1273 (m)	1331 (m)<br>1354 (m)<br>1396 (m)	1427 (vw)<br>1454 (m)<br>1467 (m)	1509 (vw)	—

[purple] = very weak (vw); [blue] = weak (w); [green] = medium (m); [orange] = strong (s); [red] = very strong (vs)

Table S7. Tentative assignments for BSA [101] [102] [103] [107] [108] [109] [110] [111].

Raman shift (cm^{-1})	Tentative assignments	Amide
640	🟩 Tyr (641), Pro (642), Met (645)	—
820	🟧 Phe (821), Tyr (830)<br>🟩 Ser (814), Ile (825)	—
850	🟧 Pro (843), Leu (848), Val (850), Ala (852), Ile (852), His (854), Ser (854)<br>🟩 Tyr (847)	—
1001	🟥 Phe (1005)<br>🟧 Ser (1010)<br>🟩 Ile (993)	—
1046	🟧 Phe (1036)<br>🟩 Tyr (1044)	—
1204	🟧 Phe (1214)<br>🟩 Val (1192), Arg (1199), Tyr (1201)	Amide III
1327	🟥 His (1319)<br>🟧 Gly (1327), Ser (1327), Ile (1329)<br>🟩 Tyr (1328), Met (1321), Arg (1331), Val (1331)	—
1448	🟧 Lys (1456)<br>🟩 Gly (1442), Arg (1443), Met (1447), Ile (1450), Val (1454), Leu (1457)	—
1605	🟩 Phe (1608), Lys (1609), Tyr (1615)	—
1670	🟩 Glu (1682)	Amide I

🟩 = medium (m); 🟧 = strong (s); 🟥 = very strong (vs)

Table S8. Tentative assignments for Ab-Gli [104] [105] [106] [107] [108] [109] [110] [111].

Raman shift (cm⁻¹)	Tentative assignments	Amide
525	🟧 Ile (536)<br>🟩 Ala (533)	—
624	🟩 Cys (613), Phe (622), Glu (623), Lys (625)	—
680	🟩 Ile (675), Cys (678), Met (682)	—
712	🟧 Met (721)<br>🟩 Ile (710)	—
855	🟧 Val (850), Ala (852), His (854), Ser (854)<br>🟩 Pro (843), Tyr (847), Leu (848), Ile (852)	—
1001	🟥 Phe (1005)<br>🟧 Ser (1010), Trp (1010)<br>🟩 Glu (988), Lys (988), Ile (993)	—
1028	🟧 Phe (1036)<br>🟩 Ile (1017), Ala (1021), Arg (1036), Gly (1036), Lys (1036)	—
1151	🟩 Ala (1149), Phe (1158)	—
1296	🟧 Lys (1305)<br>🟩 Ser (1301), Ala (1308)	Amide III
1346	🟧 Asp (1338), Cys (1341), Thr (1341), Ile (1355)<br>🟩 Lys (1341), Leu (1342), His (1349)	Amide III
1470	🟧 Ala (1464)<br>🟩 Glu (1462), Lys (1464), Ser (1464), Val (1467)	—
1509	🟩 His (1500), Ile (1514)	Amide II
1562	🟩 Trp (1559), His (1573)	Amide II
1632	🟩 Glu (1637)	Amide I
1671	🟩 Glu (1682)	Amide I

🟩 = medium (m); 🟧 = strong (s); 🟥 = very strong (vs)

Table S9. Tentative assignments for Ab-AFP [75] [107] [108] [109] [110] [111][110].

Raman shift (cm⁻¹)	Tentative assignments	Amide
506	🟥 Cys (499)<br>🟩 Gly (496)	—
683	🟩 Ile (675), Cys (678), Met (682)	—
727	🟧 Met (721)<br>🟩 His (731)	—
810	🟧 Phe (821)<br>🟩 Tyr (798), Met (805), Ser (805), His (806), Ser (814)	—
835	🟧 Tyr (830), Phe (835)<br>🟩 Ile (825), Val (825), Leu (836), Pro (843), Tyr (847)	—
880	🟥 Glu (873)<br>🟧 Thr (872)<br>🟩 Asp (873), Cys (873), Ile (873), Trp (875), Lys (877), Met (877)	—
926	🟧 Glu (917)<br>🟩 Ile (918), His (919), Pro (921), Arg (926), His (929), Thr (932)	—
988	🟧 Arg (985)<br>🟩 His (977), Tyr (986), Glu (988), Lys (988), Ile (993)	—
1045	🟧 Phe (1036)<br>🟩 Ile (1033), Arg (1036), Gly (1036), Lys (1036), Tyr (1044), Lys (1055)	—
1076	🟧 Lys (1072), Arg (1083), His (1088)<br>🟩 Val (1066), Glu (1080), Asp (1084)	—
1171	🟩 Lys (1168), His (1176), Tyr (1180), Glu (1182)	—
1196	🟩 Phe (1188), Val (1192), Arg (1199), Tyr (1201)	Amide III
1250	🟩 Pro (1239), His (1252), Ile (1257)	Amide III
1280	🟧 His (1272)<br>🟩 Val (1273)	Amide III
1417	🟩 Met (1415), Ser (1417), Thr (1419), Ile (1421), Glu (1422), Arg (1423)	—
1430	🟩 Arg (1423), Asp (1426), Trp (1426), His (1431), Lys (1433), Gly (1442)	—
1475	🟧 Ala (1464)<br>🟩 Ser (1464), Lys (1464), Val (1467), Ala (1485), Lys (1485)	Amide II
1541	—	Amide II
1566	🟩 Trp (1559), His (1573)	Amide II
1610	🟩 Phe (1608), Lys (1609), Tyr (1615), Ile (1619)	Amide I

🟩 = medium (m); 🟧 = strong (s); 🟥 = very strong (vs)

Table S10. Tentative assignments for AFP [75] [107] [108] [109] [110] [111][110].

Raman shift (cm^{-1})	Tentative assignments	Amide
500	🟥 Cys (499)<br>🟩 Gly (496)	—
681	🟩 Ile (675), Cys (678), Met (682)	—
747	🟧 Trp (756)<br>🟩 Phe (748), Asp (749), Ile (749), Val (753)	—
1206	🟧 Phe (1214)<br>🟩 Val (1192), Arg (1199), Tyr (1201), Ser (1220)	Amide III
1340	🟧 Asp (1338), Cys (1341), Thr (1341)<br>🟩 Gly (1327), Ser (1327), Tyr (1328), Ile (1329), Arg (1331), Val (1331), His (1336), Lys (1341), Leu (1342), His (1349)	Amide III
1450	🟧 Lys (1456)<br>🟩 Gly (1442), Arg (1443), Met (1447), Ile (1450), Val (1454), Leu (1457), Gly (1458), Glu (1462)	—
1530	—	Amide II

🟩 = medium (m); 🟧 = strong (s); 🟥 = very strong (vs)

Table S11. Assignments of major peaks for MPA [120] [123] [124].

Raman Shift (cm^{-1})	Band assignments
475	CCO deformation / OCO rocking
653	C–S stretching (gauche conformation)
735	C–S stretching (trans conformation)
845	CH_2 rocking
926	C–COO stretching (deprotonated form)
1058	C–C stretching
1193	CH_2 twisting
1288	CH_2 wagging
1340	CH_2 wagging
1376	symmetric COO stretching
1410	symmetric COO stretching
1462	CH_2 bending
1577	asymmetric COO stretching
1595	carboxylate-related vibration
1620	C=O stretching
